# mRNA Levels in Control Rat Liver Display Strain-Specific, Hereditary, and AHR-Dependent Components

**DOI:** 10.1371/journal.pone.0018337

**Published:** 2011-07-08

**Authors:** Paul C. Boutros, Ivy D. Moffat, Allan B. Okey, Raimo Pohjanvirta

**Affiliations:** 1 Department of Pharmacology and Toxicology, University of Toronto, Toronto, Canada; 2 Informatics and Biocomputing Platform, Ontario Institute for Cancer Research, Toronto, Canada; 3 Department of Food and Environmental Hygiene, University of Helsinki, Helsinki, Finland; 4 Laboratory of Toxicology, National Institute for Health and Welfare, Kuopio, Finland; University of Illinois at Chicago, United States of America

## Abstract

Rat is a major model organism in toxicogenomics and pharmacogenomics. Hepatic mRNA profiles after treatment with xenobiotic chemicals are used to predict and understand drug toxicity and mechanisms. Surprisingly, neither inter- and intra-strain variability of mRNA abundances in control rats nor the heritability of rat mRNA abundances yet been established. We address these issues by studying five populations: the popular Sprague-Dawley strain, sub-strains of Long-Evans and Wistar rats, and two lines derived from crosses between the Long-Evans and Wistar sub-strains. Using three independent techniques – variance analysis, linear modelling, and unsupervised pattern recognition – we characterize extensive intra- and inter-strain variability in mRNA levels. We find that both sources of variability are non-random and are enriched for specific functional groups. Specific transcription-factor binding-sites are enriched in their promoter regions and these genes occur in “islands” scattered throughout the rat genome. Using the two lines generated by crossbreeding we tested heritability of hepatic mRNA levels: the majority of rat genes appear to exhibit directional genetics, with only a few interacting loci. Finally, a comparison of inter-strain heterogeneity between mouse and rat orthologs shows more heterogeneity in rats than mice; thus rat and mouse heterogeneity are uncorrelated. Our results establish that control hepatic mRNA levels are relatively homogeneous within rat strains but highly variable between strains. This variability may be related to increased activity of specific transcription-factors and has clear functional consequences. Future studies may take advantage of this phenomenon by surveying panels of rat strains.

## Introduction

The brown Norway rat, *Rattus norvegicus*, is a major model organism for pharmacogenomic and toxicogenomic research and is widely used to assess the potential human toxicities of drugs [1], [2], [3]. Rat research also has a strong physiological focus and a long history of disease-related model systems [4], [5], although rat genetics is currently more rudimentary than mouse and other model organisms. The recent sequencing of the rat genome has increased the amount of mechanistic work done in this model [5], and has underpinned additional pharmacogenomic and toxicogenomic studies [6], [7].

An experimental system is only as valuable as our understanding of its limitations. If an experimenter cannot identify, understand, and ultimately control sources of variability, the system will not lead to robust experimental conclusions. When dealing with a model organism, it is necessary to consider the variability between members of the population as well as among different sub-populations of the same species. These features are captured in analyses of intra- and inter-strain variability amongst presumed genetically identical members of the population.

For transcriptomic studies, several groups have evaluated intra- and inter-strain variability in various model organisms. For example, the relative contributions of gender, genotype, and age to transcriptional variance in *Drosophila* have been assessed in detail [8], [9]. In mice, several studies have considered the effects of strain-to-strain variability on behaviour [10], [11]. A few analyses linking mRNA levels to sequence variation in human cell culture lines have also been performed [12], [13], [14]. Indeed, in these latter cases and a few other studies the heritability of mRNA expression profiles has also been assessed [15], [16], [17].

Surprisingly, however, these important characterizations of model organisms have not been extended to the rat; only very limited comparisons of inter-strain variability have been performed [18], [19], [20]. To estimate the intra- and inter-strain variability in mRNA abundance in rat liver, one might consider the results from a closely related species, such as mouse. A recent study of mouse liver mRNA levels demonstrated relatively high intra-strain variability, coupled to relatively low inter-strain variability across five mouse strains (3 in-bred and 2 out-bred) [21], although large inter-strain variability has also been reported [17]. If inter-strain differences are large in the rat, this would challenge the generality of the commonly applied current practice of using single rat strains for pharmacogenomic and toxicogenomic studies.

We assessed the effect of strain on mRNA expression profiles of control rat liver by surveying three strains and two lines. In striking contrast to the published mouse data, we found very large inter-strain variability. This variability is non-random: genes differentially expressed across strains are clustered in islands of the genome, are enriched for specific functional categories, and appear to be partially driven by differential transcription-factor activities. Further, we explicitly link mRNA expression to a particular allele whose variation across the five strains is known and well-characterized, the aryl hydrocarbon receptor. Expression profiles in rat liver are highly heritable, with the vast majority of genes displaying directional genetics. Finally, the genes that display inter-strain variability are different in rat than in mouse.

## Results

To evaluate intra- and inter-strain variability in mRNA abundances in the rat we assessed hepatic mRNA levels in control animals (corn oil treated) from three (sub-) strains and two lines of rat. We chose to focus on liver because of its relatively low degree of cellular heterogeneity and its importance in drug and xenobiotic metabolism. The three strains selected were Sprague-Dawley (S-D, out-bred), Long-Evans (*Turku/AB*) (L-E, in-bred) and Han/Wistar (*Kuopio*) (H/W, random-bred/closed-colony). S-D and the background strains of L-E and H/W are amongst those most widely used in biomedical research. The two lines are descendants of L-E×H/W crosses, and are termed Line-A and Line-C [22]. Corn oil is expected to have minimal effects on basal hepatic mRNA abundances, but was included in our experiments to mimic actual pharmacological and toxicological studies where it is a frequently used solvent for lipophilic compounds. We carefully validated the quality and performance of our microarray data (Figures S1, S2, S3, and see below).

**Figure 1:**
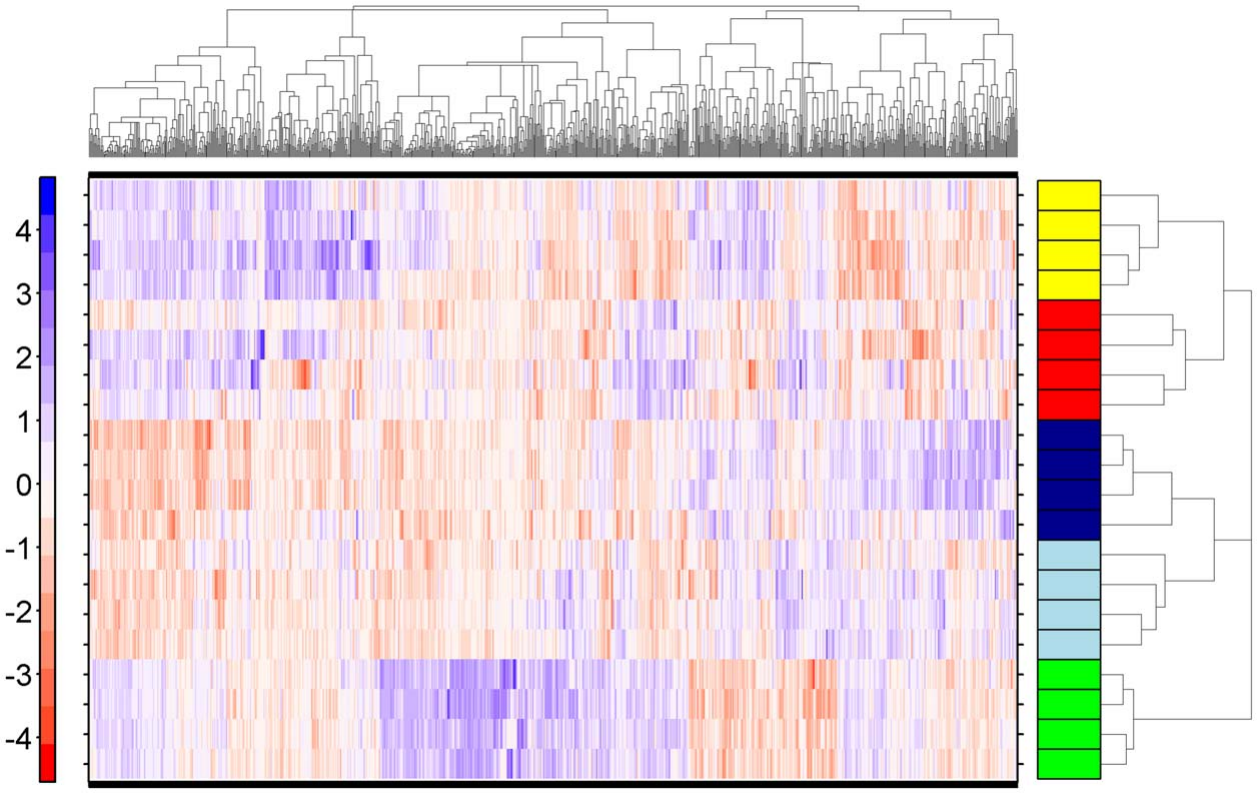
mRNA Abundances Across Five Rat Strains and Lines. The hepatic mRNA abundance profiles of five rat strains and lines were determined using microarray methods. Following pre-processing, the variance of each ProbeSet was calculated: those having a variance above 0.25 were subjected to divisive hierarchical clustering using the DIANA algorithm. Data were mean-centered and root-mean-square-scaled prior to clustering. Columns are genes, rows are individual animals. The colour-bar for the rows indicates the strain or line of that animal. Yellow, Long-Evans; Red, Han/Wistar; Dark Blue, Line-A; Light Blue, Line-C; Green, Sprague-Dawley.

For each strain or line, we assessed hepatic mRNA expression in four independent animals. We selected those genes that are most variable and subjected them to pattern-recognition [23]. This unsupervised machine-learning analysis perfectly separated the five strains/lines (Figure 1). Importantly, the two lines clustered together. Thus a simple and unbiased analysis shows profound inter-strain differences in steady-state hepatic mRNA levels.

### Analysis of Intra-Strain Variability

To assess intra-strain variability, we performed a variance analysis on our transcriptome-wide array data. For each array feature we calculated the within-strain (W) and between-strain variances (B) using a mixed model. We calculated the total variance (T = W+B) and used the ratio W/T as an unbiased estimator of intra-strain variability in mRNA abundances. This type of analysis has been applied to the study of cancer [24], but to our knowledge this is its first application to the genetics of gene expression. Our complete W/T results are given in Table S1.

**Figure 2:**
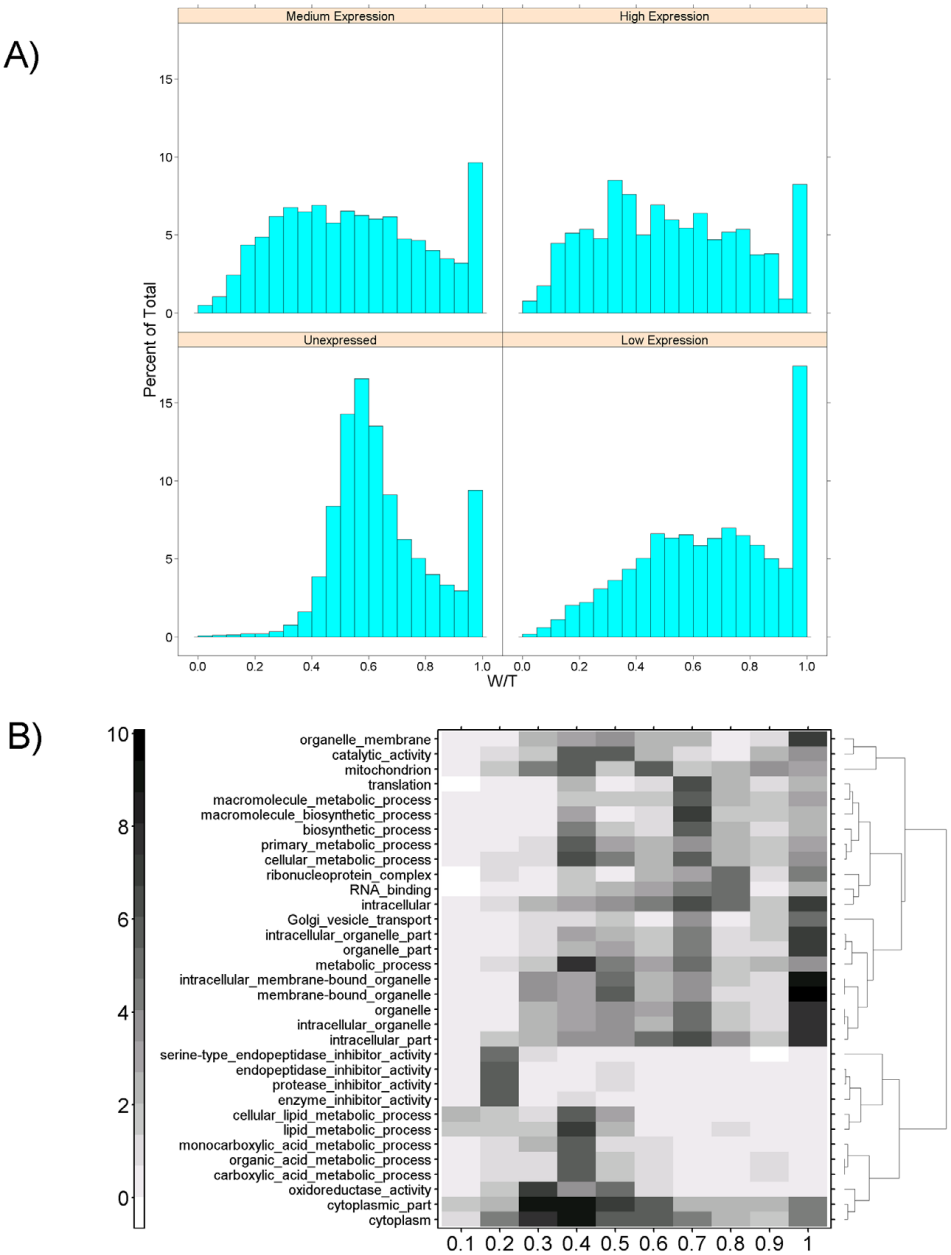
Intra-Strain Variability in mRNA Abundance. To assess the variability of mRNA abundances within and between strains of rats we performed a variance analysis using a mixed model. For each ProbeSet, we calculated the variance between strains (B), the variance within strains (W) and, from these, the total variance (T = W+B). The ratio W/T shows how much of the variability in signal is related to strain-assignment and how much is related to individual variability. A W/T value of 1 indicates high within-strain variability, while a value of 0 indicates high inter-strain variability. A) Histograms showing the distribution of W/T values at four different mRNA levels calculated for each strain show a generally unimodal distribution with a secondary peak with high W/T values. The distribution generally flattens as expression levels rise. B) ProbeSets were divided into ten groups based on their W/T values, and subjected to Gene Ontology enrichment analysis. All GO terms enriched at p<10^−5^ in at least one group were selected and subjected to divisive hierarchical clustering. Rows represent GO terms, columns represent groups with specific W/T ranges, and the individual cells in the heatmap represent the −log_10_ of the p-value for enrichment of the GO term in that group.

We analyzed the distribution of W/T values at different expression levels within each strain by dividing ProbeSets into four groups based on their normalized signal intensity: <4 (unexpressed), 4–8 (low level of expression), 8–12 (medium level of expression), and >12 (high level of expression). Histograms for each of these groups (Figure 2A) show a unimodal distribution centred near 0.5 (indicating equal intra-strain and inter-strain variability), combined with a sharp peak near 1.0 (indicating all variance is within strains).

Previous analyses of cancer samples [24] showed that W/T values were tightly associated with gene function. Thus we tested the hypothesis that W/T values are associated with specific functional groups by performing Gene Ontology enrichment analysis using the GOMiner tool. We divided ProbeSets into ten equally-spaced groups, based on W/T values ranging from 0 to 1, and performed GO enrichment analysis on each. We selected all GO terms strongly enriched in at least one group (p<10^−5^) and subjected them to divisive hierarchical clustering (Figure 2B). Several trends are evident: protease inhibitors tend to have low W/T values, lipid-metabolizing genes tend to have intermediate ones, and RNA-binding genes tend to have high ones. We note that the number of genes in each interval differs slightly, creating differences in power that are unaccounted for in this analysis. Our complete analysis relating W/T to Gene Ontology terms is given in Table S2.

### Analysis of Inter-Strain Variability

**Figure 3:**
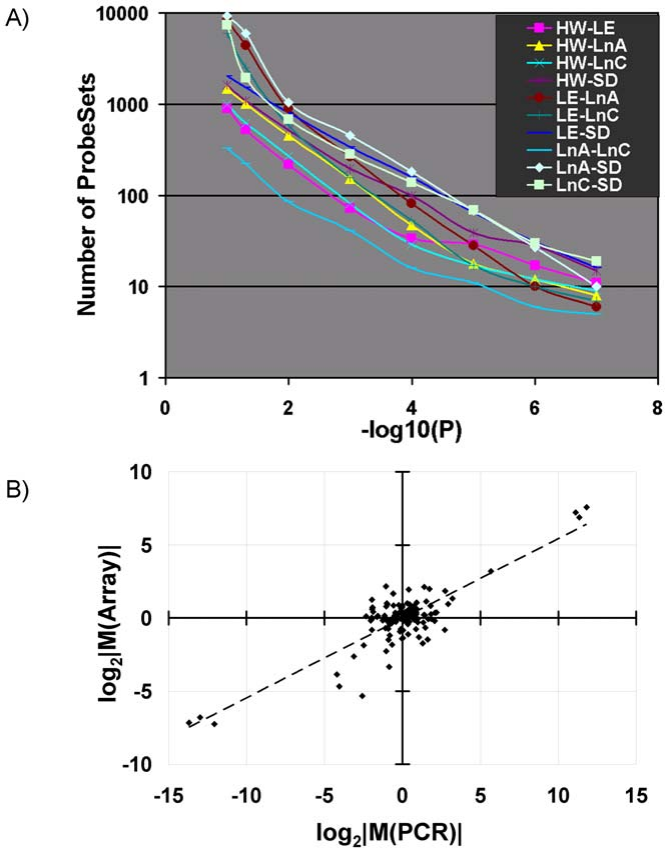
Pair-Wise Comparisons of Rat Strains/Lines. A) Linear-modelling was used to identify differentially-expressed ProbeSets between all ten pairs of rat strains/lines. The number of ProbeSets differentially expressed for each pair (y-axis) is plotted as a function of the P-value threshold (x-axis). The ranking of different pairs remains consistent, independent of the threshold selected. B) To validate our analysis 21 genes were also assessed using gold-standard real-time RT-PCR. For each gene all pair-wise comparisons were made and the fold-changes calculated. This led to 131 comparisons that were made by both RT-PCR and microarray. When these are plotted in log_2_-space it is clear that the two assays yield highly correlated measurements (Pearson's R = 0.839; p<2.2×10^−16^).

Our variance analysis suggested that most genes expressed in control rat liver showed greater inter-strain variability than intra-strain variability. To quantify the inter-strain variability we employed a linear-modelling analysis [25]. We compared the mRNA levels of all ten pairs of strains/lines: complete data for all ProbeSets are available in Table S3. At a false-discovery rate of 1%, the average pair of strains had 558±311 differentially-expressed ProbeSets. Importantly the two descendants of L-E×H/W crosses (Line-A and Line-C) were the most similar pair, with only 85 ProbeSets differentially expressed between them. The rankings of strain-to-strain variability were consistent across a broad range of FDR thresholds (Figure 3A). To confirm the unsupervised analysis described above, we filtered ProbeSets based on the F-statistic from the linear-model fit and performed clustering (Figure S4). An identical clustering profile was observed regardless of whether ProbeSets were selected based on overall variance filter or on the F-statistic. Thus our unsupervised analysis, our variance analysis, and our statistical analysis all confirm that inter-strain variability exceeds intra-strain variability by a large margin.

To validate our high-throughput results, we embarked on extensive validation by real-time PCR. We selected 21 genes for validation, selected to have a wide-range of differential expression and mRNA abundance. For each of these genes, we assayed its mRNA levels in three or more strains, using between three and six animals for each strain. In total, then, we performed 366 RT-PCR validations. We calculated all pair-wise fold-changes and compared them to the fold-changes calculated from the microarray data (Table S3). We plotted the array and PCR results against one another (Figure 3B) and observed a high correlation between the two assays (Pearson's R = 0.839, p<2.2×10^−16^).

### Analysis of AHR-Dependent Variability

The previously published rat genome sequence was derived from a highly inbred sub-strain of Brown Norway rat [26] and, to our knowledge, no genome-wide SNP or copy-number analyses of the five rat strains/lines we studied exist. If such datasets were available it would be possible to attempt to link specific genomic features with variations in mRNA profiles. In the absence of such a genome-wide sequence analysis, however, there is one specific locus known to differ across the five strains/lines: the aryl hydrocarbon receptor (*Ahr*).

It has been well-established that the *Ahr* regulates induction of multiple drug-metabolizing enzymes, mediates dioxin toxicity [27], and plays important developmental roles [28], [29]. One of the three strains used here, Han/Wistar, bears a mutant *Ahr* [30]. This *Ahr* variant leads to a dramatic resistance to dioxins, but with no apparent developmental defects. While one of the lines (Line-A) also bears this mutant *Ahr*, the other (Line-C) and the Long-Evans and Sprague-Dawley strains harbour wild-type AHR proteins.

To assess the global effect of this variant AHR on mRNA levels in rat liver we performed a second linear-modelling analysis. Each of the five strains/lines was modelled as having strain-specific and AHR-specific components. Many ProbeSets appeared to show *Ahr*-variant specific expression patterns: 15 ProbeSets showed an effect of the variant *Ahr* at a 0.1% FDR, 42 ProbeSets were affected at 1% FDR, and 105 ProbeSets were affected at 5% FDR (Table 1 and Table S4). This finding is particularly striking given the fact that the Line-A and Line-C strains have nearly identical transcriptomes (Figures 1 & 3, and Figures S1 & S4).

**Table 1:** Selected ProbeSets Showing AHR-Dependent mRNA Levels.

ProbeSet	Symbol	Entrez Gene ID	M	Q	Gene Title
1384240_at	Agtr1a	24180	6.9	3.76×10^−15^	angiotensin II receptor, type 1 (AT1A)
1369291_at	Agtr1a	24180	5.8	4.66×10^−13^	angiotensin II receptor, type 1 (AT1A)
1381968_at	Creg_predicted	289185	−2.3	4.01×10^−7^	cellular repressor of E1A-stimulated genes (predicted)
1372342_at	Mrvldc1	309375	5.9	3.14×10^−6^	MARVEL (membrane-associating) domain containing 1
1368826_at	Comt	24267	−2.9	6.44×10^−6^	catechol-O-methyltransferase
1387981_at	Olr59	170816	3.6	3.91×10^−5^	olfactory receptor 59
1376796_at	Rab14	94197	1.1	2.38×10^−4^	RAB14, member RAS oncogene family
1387144_at	Itga1	25118	−3.3	4.38×10^−4^	integrin alpha 1
1368171_at	Lox	24914	6.7	1.27×10^−3^	lysyl oxidase
1367593_at	Sepw1	25545	−2.3	1.77×10^−3^	selenoprotein W, muscle 1
1372925_at	Sirt3_predicted	293615	2.3	2.00×10^−3^	sirtuin 3 (silent mating type information regulation 2, homolog) 3 (S. cerevisiae) (predicted)
1368172_a_at	Lox	24914	3.4	2.35×10^−3^	lysyl oxidase
1368155_at	Cyp2c12	25011	−3.9	8.72×10^−3^	cytochrome P450, family 2, subfamily c, polypeptide 12
1367609_at	Mif	81683	1.2	1.34×10^−2^	macrophage migration inhibitory factor
1375936_at	Dsc2	291760	−2.9	1.91×10^−2^	desmocollin 2
1388917_at	Myo1d	25485	−1.0	2.02×10^−2^	myosin ID
1370154_at	Lyz	25211	−1.8	2.86×10^−2^	lysozyme
1367988_at	Cyp2c23	83790	−0.5	3.41×10^−2^	cytochrome P450, family 2, subfamily c, polypeptide 23
1370698_at	Udpgtr2	286954	1.4	5.11×10^−2^	liver UDP-glucuronosyltransferase, phenobarbital-inducible form

Following pre-processing, a linear-modelling approach was used to identify ProbeSets associated with AHR status. A subset of these is shown here. The M-values represent the magnitude of difference in expression caused by the mutant AHR in log_2_ space. For example, 1368826_at has an M-value of −2.9, indicating a 7.5-fold repression in signal intensity in rats that harbour the variant AHR. The column Q gives the false-discovery rate (a multiple-testing adjusted p-value).

**Figure 4:**
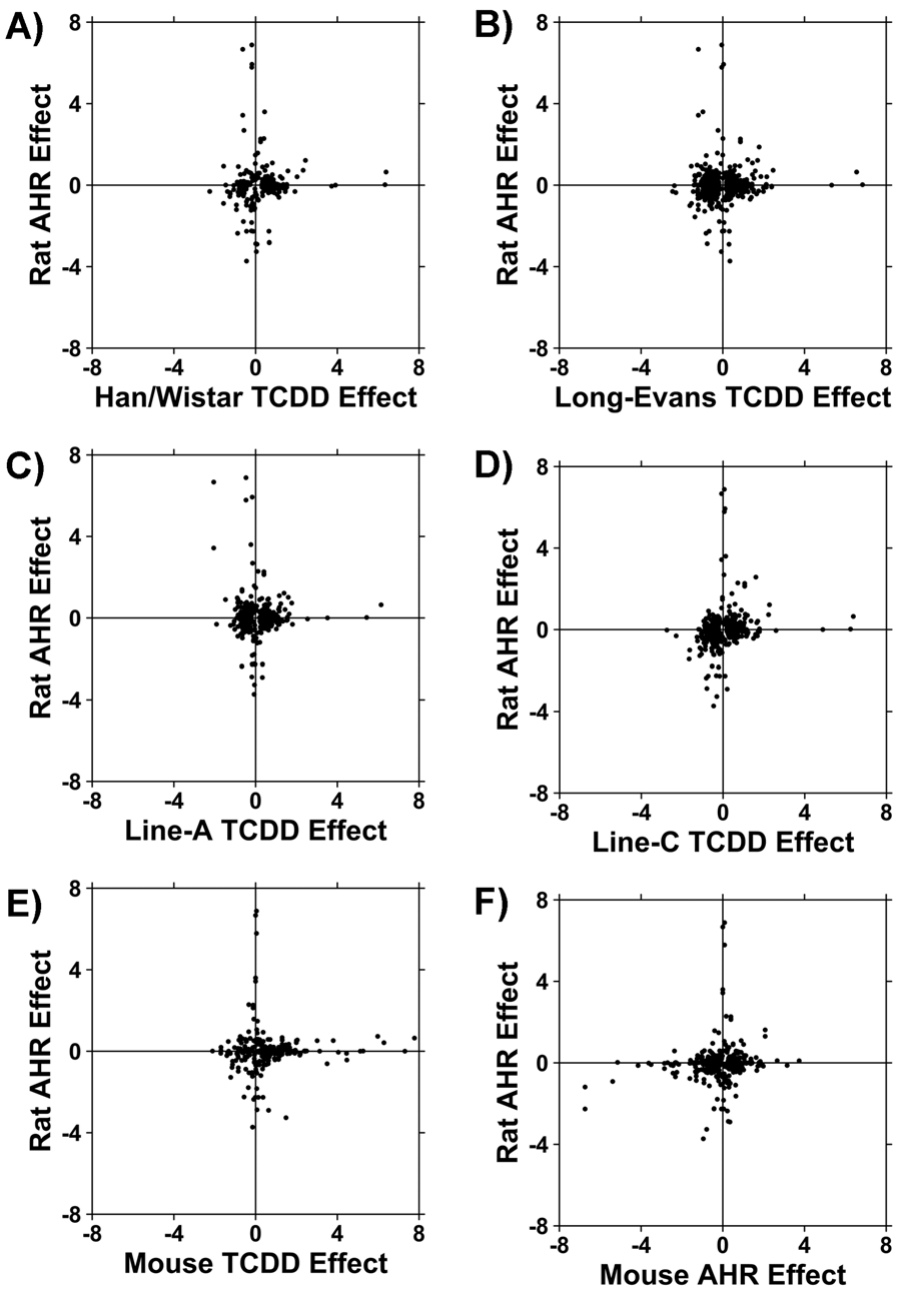
Comparison of *Ahr* Effect in Other Datasets. We next studied the set of genes whose mRNA levels were different between rats harbouring the wildtype *Ahr* allele (i.e. Long-Evans, Sprague-Dawley, and Line-C rats) and those harbouring the mutant *Ahr* ^H/W^ allele (i.e. Han/Wistar and Line-A). We examined the response of these genes to a potent AHR ligand, TCDD, in four rat strains, in wildtype C57/BL6J mice, and in *Ahr* ^−/−^ mice. A) The effects of the *Ahr* ^H/W^ genotype in control rats were uncorrelated to the effects of TCDD in H/W rats (R = 0.034, P = 0.60) B) The basal effects of the *Ahr* ^H/W^ genotype in rats were uncorrelated to those of TCDD in L-E rats (R = 0.036, P = 0.26) C) The basal effects of the *Ahr* ^H/W^ genotype in rats were uncorrelated to those in Line-A rats (R = −0.064, P = 0.16) D) The basal effects of the *Ahr* ^H/W^ genotype in rats were weakly, but statistically significantly, correlated to those in Line-C rats (R = 0.19, P = 3.3×10^−6^) E) The basal effects of the *Ahr* ^H/W^ genotype in rats were uncorrelated to the effects of TCDD in C57BL/6J mice (R = 0.026, P = 0.65). F) The basal effects of the *Ahr* ^H/W^ genotype in rats were weakly, but statistically significantly, correlated to the effects of genetic ablation of the *Ahr* locus in mice (R = 0.14, P = 0.0035).

Focusing on the 105 ProbeSets affected by *Ahr* genotype at 5% FDR, we interrogated their response to stimulation by AHR ligands. We compared these 105 ProbeSets to a study of alterations in mRNA abundances in four strains of rats induced by TCDD (2,3,7,8-tetrachlorodibenzo-*p*-dioxin), a potent AHR ligand [31]. In all H/W, L-E, and Line-A rats there was minimal relationship between the genes induced by TCDD (Figure 4A–C), with correlations ranging from −0.06 to +0.03. In Line-C rats (Figure 4D). However, there was a significant association, with a modest, but statistically-significant correlation of 0.19 (p = 3.3×10^−6^). We then looked at genes stimulated by TCDD in mouse liver [32] to see if there was any cross-species conservation and, again, found no correlation (Figure 4E).

These data suggested that the vast majority of genes basally affected by AHR genotype differ from those stimulated by exogenous ligands – a similar conclusion to that reached by study of liver [33] and kidney [32] of *Ahr* ^−/−^ mice. We thus compared the rat genes to those mouse genes affected by ablation of the *Ahr* locus. We observed a small, but statistically significant correlation of 0.14 (p = 0.0035, Figure 4F). This small, but non-random overlap between mouse and rat at the basal level mimics observations in TCDD-exposed animals [34], [35]. Four genes showed statistically significant changes in both species: branched chain keto acid dehydrogenase E1 (up in rat, down in mouse), glyoxalase 1 (up in both species), beta ureidopropionase (down in both species), and GrpE-like 1 (down in both species).

**Figure 5:**
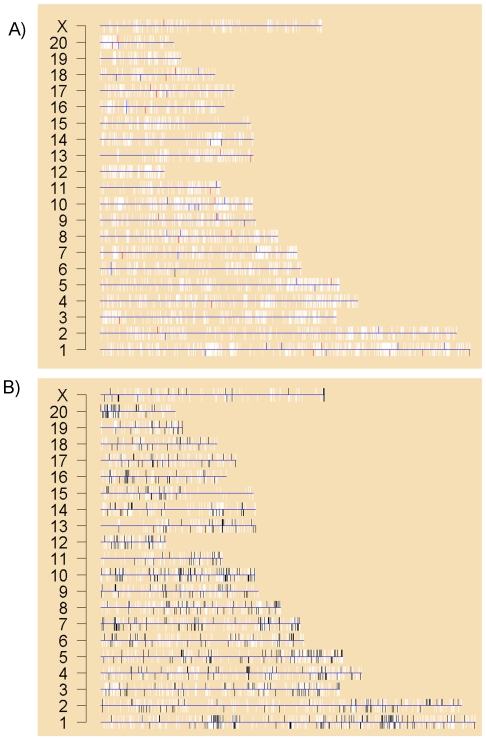
Genome-Wide Mapping of Differential Expression. To determine if differentially-expressed ProbeSets were localized to specific portions of the rat genome we plotted the entire genome, with one chromosome per line. Each gene was plotted with a white bar representing its location on the chromosome and its position on the plus (up) or minus (down) strand. A) Genes showing AHR-dependent expression have been colour-coded in red (down-regulated) or blue (up-regulated). The AHR itself is in black on chromosome 6. B) Genes displaying strain-specific mRNA abundances were identified by using a linear model and selected ProbeSets where the F p-value<0.001. These genes are plotted in black, and form clear clusters throughout the genome.

Because the Han/Wistar and Line-A rats that bear the mutant *Ahr* are very similar genetically, it is theoretically possible that the *Ahr*-dependent expression patterns are artifacts of recombination events. If this were the case we would expect to see these concentrated into islands within the genome. When we mapped these 105 ProbeSets onto the genome no such islands were observed (Figure 5A).

We employed the same approach to assess if genes that show inter-strain variability were concentrated into specific regions of the genome. We selected those ProbeSets showing significant inter-strain variability in the pair-wise analysis (p<0.001 based on the F-statistic) and mapped them onto the rat genome (Figure 5B). Putative “hotspots” are evident on chromosomes 1, 4, 7, 10, 13, 16, and 20. Previously, in mouse, we found that genes whose mRNA levels are influenced by *Ahr* status alone as well as genes that respond to AHR activation by dioxin are widely dispersed across the genome with only modest clustering into “hotspots” [33].

### Analysis of Function by Gene Ontology

The above analyses identified numerous genes whose mRNA levels are strain-specific or AHR-dependent by varying degrees of magnitude. It is important to know if genes that show strain-dependent differences in mRNA abundance represent a random selection of the genome or are biased towards specific functional pathways. If specific functions are enriched this would imply that the results of pharmacogenomic and toxicogenomic studies will depend on strain-selection. For genes exhibiting *Ahr*-dependent mRNA levels, functional enrichment can shed light on the mechanism by which the aberrant *Ahr* isoform protects animals from dioxin toxicity.

**Figure 6:**
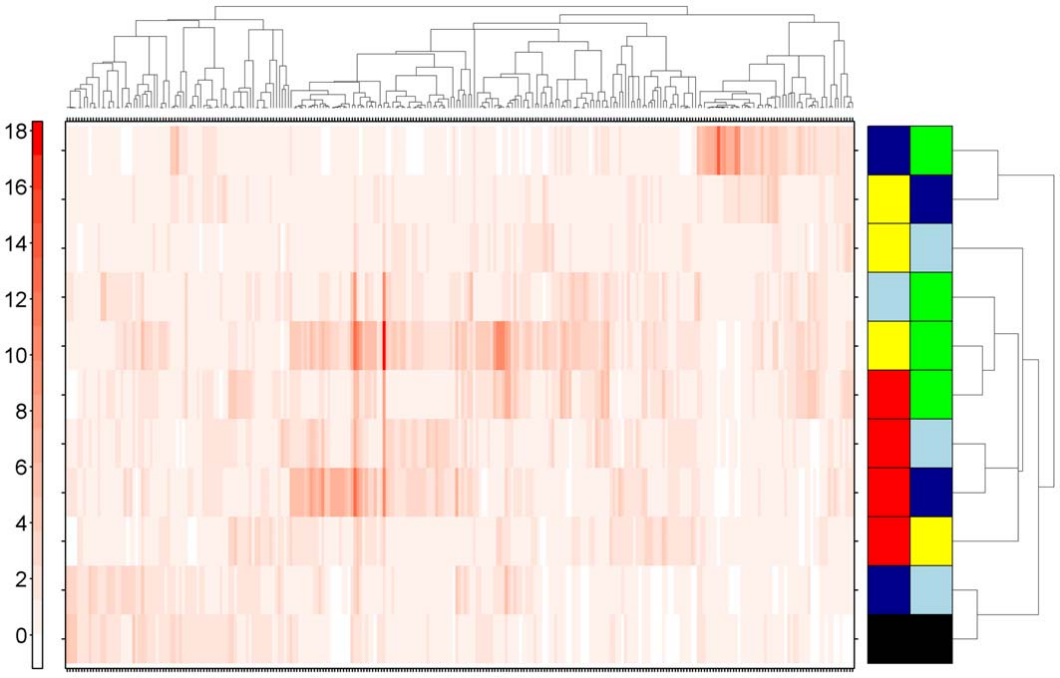
Gene Ontology Analysis of Differentially-Expressed Genes. To identify functional trends and similarities amongst pairs of strains, we again employed Gene Ontology enrichment analysis. Those genes differentially expressed (p_adjusted_<0.05) in each strain were used, and only those GO terms significantly enriched at a cumulative probability of p_cumulative_<10^−10^ were selected. The p-values for these selected GO terms were subjected to divisive hierarchical clustering, with the contrasts as rows, the GO terms as columns, and the colour of individual cells indicating the −log_10_ of the p-value for enrichment. The two colour bars on the right side of the figure indicate the two strains compared in this contrast. Yellow, Long-Evans; Red, Han/Wistar; Dark Blue, Line-A; Light Blue, Line-C; Green, Sprague-Dawley; Black, AHR.

To address these questions at a genome-wide scale we employed gene-ontology enrichment analysis [36] for each pair-wise comparison amongst strains, as well as for genes exhibiting *Ahr*-dependent expression. We identified the most enriched GO terms across all eleven conditions by summing the log_10_|P| values and selecting terms with a cumulative score below −10 (i.e. an unadjusted cumulative enrichment probability of 10^−10^). We subjected these terms to divisive hierarchical clustering (Figure 6).

This clustering analysis has three salient features. First, AHR-specific GO terms and GO-terms generated from the Line-A vs. Line-C pair-wise comparison cluster closely together. This is reassuring, since the two lines should be highly similar outside of their AHR loci because of their common origin in L-E×H/W crosses [22]. Second, Han/Wistar (red) displays a consistent difference from the other strains/lines, as demonstrated by their co-clustering. Third, the most distant cluster contains the comparison of the dioxin-resistant Line-A rats with the two dioxin-sensitive strains, Sprague-Dawley and Long-Evans.

To confirm that our findings are independent of the p-value threshold of 10^−10^, we repeated this analysis using GO terms with cumulative probabilities below 10^−5^ (Figure S5), 10^−7.5^ (Figure S6), 10^−20^ (Figure S7), and 10^−30^ (Figure S8).

Having examined the global perturbation of pathways across different strains, we next considered the specific GO terms enriched in these analyses. Selected results are in Table 2; the complete GO analysis is in Table S5. Some functional groups are specifically altered in only some pair-wise comparisons. For example, calcium ion binding (GO:0005509) genes are differentially expressed between S-D and LnA rats (p = 3.89×10^−8^) but not in any other pair-wise comparisons. Other functions are differentially altered in one strain relative to all others, such as oxidoreductase activity (GO:0016491) which is differentially expressed in H/W rats relative to the other four strains/lines (p<1.05×10^−4^ for all four pair-wise comparisons). Finally, and perhaps most interestingly, genes exhibiting *Ahr*-dependent mRNA abundance are enriched for those involved in cholesterol absorption (p = 1.05×10^−5^).

**Table 2:** Selected Enriched Gene Ontology Categories.

GO ID	AHR	HW	HW	HW	HW	LE	LE	LE	LnA	LnA	LnC	GO Term
		LE	LnA	LnC	SD	LnA	LnC	SD	LnC	SD	SD	
GO:0005509	−0.04	0.00	−0.02	−0.01	−0.01	−1.64	−0.09	−0.30	−0.01	−7.41	−0.33	calcium ion binding
GO:0005737	−1.40	−3.55	−10.07	−7.80	−7.38	−1.67	−3.49	−17.14	−2.17	−0.08	−10.07	cytoplasm
GO:0005739	−2.49	−1.02	−6.52	−3.66	−2.21	−0.56	−0.84	−6.54	−2.41	−0.10	−3.96	mitochondrion
GO:0005770	−0.78	−4.67	−0.57	−2.40	−4.37	−0.28	−0.11	−1.88	−0.52	−0.92	−2.71	late endosome
GO:0005886	−0.28	−0.23	0.00	−0.09	−0.54	−0.47	−0.03	−0.04	−0.03	−8.46	−0.64	plasma membrane
GO:0006082	−0.91	−2.66	−0.93	−0.30	−3.52	−0.91	−0.43	−9.95	−1.43	−0.01	−1.03	organic acid metabolic process
GO:0006629	−2.31	−2.32	−3.37	−1.50	−7.18	−0.92	−1.92	−7.95	−5.16	−0.08	−1.87	lipid metabolic process
GO:0008202	−1.05	−0.52	−2.03	−1.23	−4.10	−1.10	−1.96	−4.64	−3.60	−0.03	−3.07	steroid metabolic process
GO:0009060	0.00	−0.50	−7.30	−0.40	−0.39	−0.36	−0.13	−2.44	−0.44	−0.41	−0.48	aerobic respiration
GO:0016491	−2.10	−4.83	−8.63	−3.98	−5.07	−0.51	−1.37	−10.42	−3.72	−0.04	−2.42	oxidoreductase activity
GO:0019882	−0.52	−0.98	−1.22	−3.65	−5.12	−0.37	−1.14	−5.29	−0.29	−1.59	−3.29	antigen processing and presentation
GO:0030300	−4.98	0.00	−2.03	−1.48	0.00	−0.23	−1.12	−0.26	−4.08	−0.34	−1.39	regulation of cholesterol absorption
GO:0048037	−1.40	−0.67	−6.40	−1.59	−3.11	−0.71	−0.51	−5.04	−4.32	−0.09	−1.94	cofactor binding
GO:0048731	−0.05	−0.03	0.00	−0.11	−0.01	−1.96	−0.86	−0.01	−0.01	−9.95	−0.41	system development

ProbeSets differentially expressed between strains/lines or showing AHR-dependent effects were identified using linear models and subjected to Gene Ontology enrichment analysis to identify specific pathways or functions modulated. A selection of enriched GO terms is shown. The numeric values are log_10_ P-values for enrichment of the GO term. For example, a value of −3 indicates a 0.001 probability that the observed enrichment occurred by chance alone. Each column corresponds to a separate contrast, either of two strains (e.g. H/W vs. L-E or L-E vs. S-D) or of the AHR-dependent genes.

### Analysis of Transcription-Factor Binding-Site Enrichment

Our microarray analyses identified both inter-strain and AHR-dependent trends in mRNA expression profiles. The Gene Ontology analysis outlined above suggests that these trends are functionally coherent: specific biological pathways or functions were enriched, while others were not. We hypothesized that the most probable mechanism underlying this occurrence would be differential activity of specific transcription-factors. To test for this differential occurrence we performed an analysis of transcription-factor binding-sites (TFBSs). We analyzed the promoters of genes showing strain- or *Ahr*-dependent mRNA expression for enrichment or depletion of the sequence motifs for 123 different site-specific DNA-binding proteins. For each TFBS, our analysis determined the probability that this site was enriched or depleted amongst genes whose mRNA abundance showed *Ahr*- or strain-dependency.

The results from this analysis (Table 3) indicate that nine separate TFBSs are enriched in one or more contrast. As with the analysis of GO functions, some effects were strain-specific, while others were more general. One prominent strain-specific effect is the enrichment of putative ID1 target genes differentially expressed in Long-Evans relative to Line-A, Line-C, and Sprague-Dawley (p = 0.003 for each comparison). A more general effect is enrichment of AP2 binding-sites in eight of the eleven strain pairs. Two TFBSs were enriched in genes with *Ahr*-dependent abundances: AP2 and NR2F1 (COUP-TF1).

**Table 3:** Analysis of TFBS Enrichment.

Motif ID	AHR	HW	HW	HW	HW	LE	LE	LE	LnA	LnA	LnC	Motif Name
		LE	LnA	LnC	SD	LnA	LnC	SD	LnC	SD	SD	
MA0003	0.007	<0.001	NS	<0.001	<0.001	<0.001	<0.001	<0.001	<0.001	<0.001	<0.001	TFAP2A AP2
MA0017	0.002	NS	NS	NS	NS	NS	NS	NS	NS	NS	NS	NR2F1 NUCLEAR RECEPTOR
MA0034	0.998	NS	NS	NS	NS	NS	NS	NS	0.998	NS	NS	GAMYB TRP-CLUSTER
MA0049	NS	<0.001	<0.001	<0.001	NS	<0.001	<0.001	<0.001	NS	<0.001	<0.001	Hunchback ZN-FINGER, C2H2
MA0057	NS	0.022	0.038	0.010	<0.001	<0.001	<0.001	<0.001	NS	<0.001	NS	ZNF42_5-13 ZN-FINGER, C2H2
MA0065	NS	NS	NS	<0.001	NS	NS	NS	NS	NS	NS	NS	PPARG-RXRA NUCLEAR RECEPTOR
MA0074	<0.001	NS	NS	NS	NS	NS	NS	NS	NS	NS	NS	RXR-VDR NUCLEAR RECEPTOR
MA0115	NS	NS	<0.001	<0.001	NS	<0.001	<0.001	NS	NS	<0.001	<0.001	NR1H2-RXR NUCLEAR RECEPTOR
MA0120	NS	NS	NS	NS	NS	0.003	0.003	0.003	NS	<0.001	<0.001	ID1 ZN-FINGER, C2H2

ProbeSets differentially expressed between strains/lines or showing AHR-dependent effects were identified using linear models. The promoter regions for these genes were extracted from UCSC build rn4 of the rat genome and analyzed for transcription-factor binding-site (TFBS) enrichment using a library of 123 position-weight matrices. Statistical significance was estimated using five separate tests, and only those matrices enriched in at least four of the five are reported here. The Motif ID is the identifier given in the JASPAR library, and the numeric columns correspond to the p-value for enrichment (close to zero) or depletion (closer to one) of that motif in each contrast. The p-values reported here are from 1,000 permutations of a background dataset of hepatically expressed genes (see Methods). NS, not significant (p<0.05).

### Heritability of mRNA Abundance

The complex inheritance of quantitative traits can occur in several ways. If the phenotypes of the offspring lie between the extremes of the two parents, then the trait is said to display directional genetics. If, on the other hand, the phenotype shows more extreme values in the offspring than in the parents, then the trait is said to display interacting loci. Because our study contains the mRNA expression profiles of two lines descended from L-E×H/W crosses, along with those of the parental strains, we could distinguish between these two possibilities for each ProbeSet on the array.

**Figure 7:**
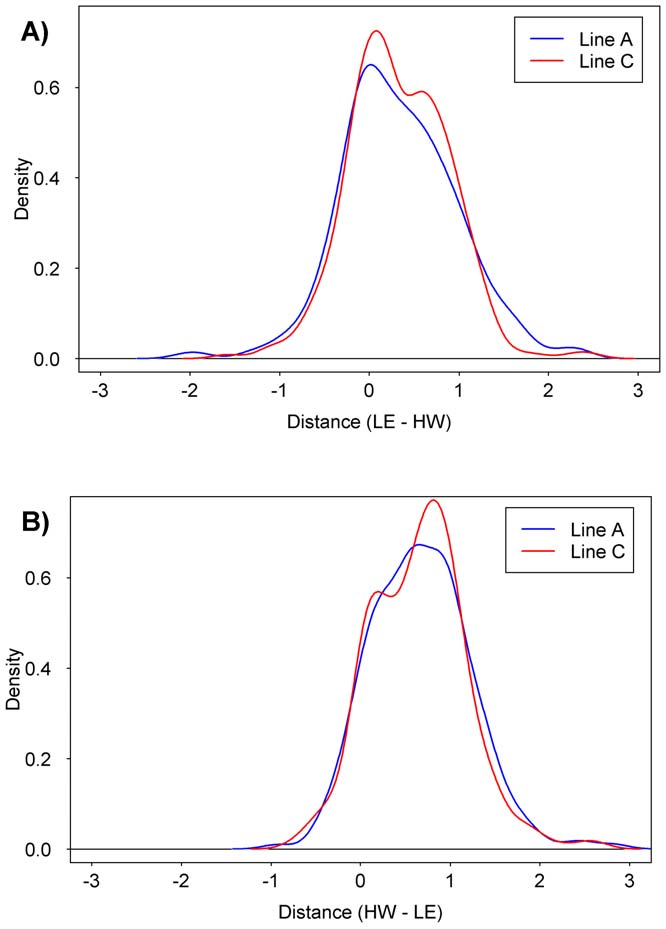
Heritability of mRNA Abundances. To analyze the heritability of mRNA abundances we used the two parental strains (L-E, H/W) and two lines resulting from L-E×H/W crosses (LnA, LnC). For each ProbeSet on the array we calculated where the Line-A and Line-C expression levels lie relative to the two parental strains. Values of zero indicate equivalent expression to the lower of the two parental strains, while values of one indicate equivalent expression to the higher of the two parental strains. A) Gaussian density plots of expression distances for Line-A and Line-C rats for ProbeSets where L-E expression is higher than H/W expression. B) Gaussian density plots of expression distances for Line-A and Line-C rats for ProbeSets where L-E expression is lower than H/W expression.

To analyze the heritability of mRNA expression across all loci, we calculated separately where the mRNA expression profiles of the Line-A and Line-C progeny lie relative to the two parental strains. Distance values of 0.0 indicate that mRNA levels in the line are equivalent to those in the lower-abundance strain; values of 1.0 indicate levels equivalent to the higher-abundance strain. If the distance is less than 0 or greater than 1, then the mRNA levels of the line lie outside the two parental strains. We plotted the results separately for ProbeSets with greater L-E signal than H/W signal (Figure 7A) and those where H/W signal is greater than L-E signal (Figure 7B). In each case a very strong peak between 0 and 1 was observed, with few extreme outliers. These results indicate that the majority of ProbeSets follow directional genetics. A third phenomenon, transgressive segregation, could not be evaluated here because only two crosses were available.

To rigorously identify outliers than might contain evidence of interacting loci, we searched our pair-wise linear model for ProbeSets that displayed statistically significantly more extreme signals in either of the two lines than in the two parents. In total, 41 ProbeSets were found to have genetic interactions, including 7 found in both Line-A and Line-C (*Asgr2*, *Ctnnb1*, *Galt*, *Garabarapl2*, *RGD1311563*, *Slco2a1*, and an expressed locus). The full set of interacting loci is given in Table S6.

### Cross-Tissue Conservation of Inter-Strain Variability

**Figure 8:**
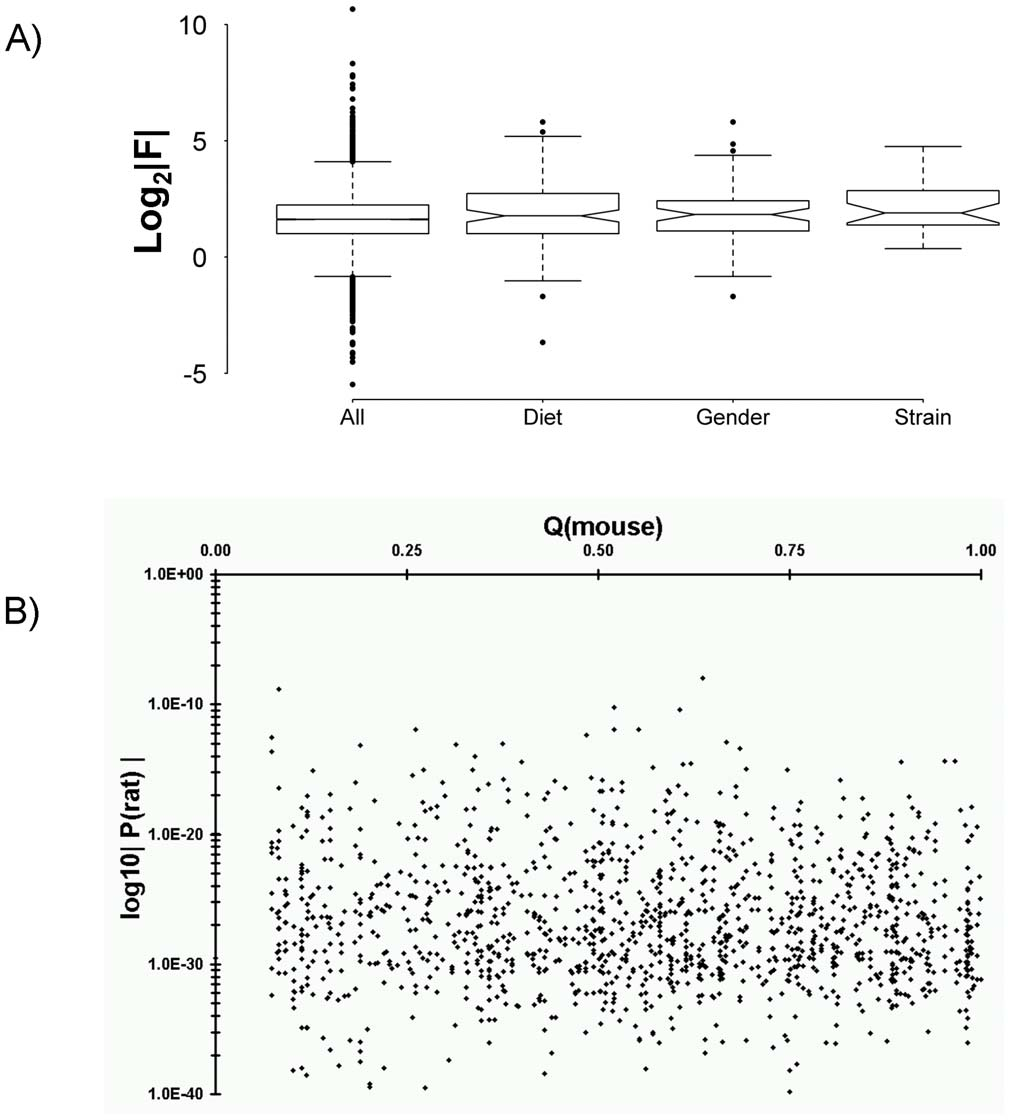
Comparison of Inter-Tissue and Inter-Species Variability. A) We compared inter-strain variability in mRNA abundances between different rat tissues by using a public dataset dataset of inter-strain variability in kidney of two rat strains. We reannotated their data to UniGene build Rn.171. The kidney study grouped genes into four categories: all genes (All), genes showing diet-dependent mRNA abundances (Diet), genes showing gender-dependent mRNA abundances (Gender), and genes showing strain-dependent mRNA abundances (Strain). The F-statistics for genes in each of these four groups were log_2_-transformed and summarized in box-plots. While genes showing a strain-dependence in kidney showed a trend towards strain-dependence in liver, this was not statistically significant (p>0.05). B) We compared genes that have strain-dependent hepatic mRNA abundances in rat to those with similar characteristics in mice by using a public dataset of five mouse strains. We reannotated their data to UniGene build Mm.168 and employed build 58 of the Homologene database to identify mouse and rat ortholog pairs. For each ortholog pair identified, we plotted the unadjusted P-value for rat inter-strain variability (y-axis) against the published q-value for mouse inter-strain variability (x-axis). No association between the two metrics is observed (Spearman's rho = 0.04, p = 0.22).

We wondered if genes that showed strain-dependent mRNA levels in liver would be similar or different from those that showed strain-dependent mRNA levels in other organs or tissues. As a simple way of analyzing this effect we used a public dataset of rat genes that showed strain-dependent abundances in kidney of two strains: Sprague-Dawley and Fischer 344 [18]. We took their dataset, mapped the GenBank identifiers to UniGene build Rn.171 and extracted the F-statistics from our linear model. Where multiple ProbeSets corresponded to a single gene, we made no assumptions about which ProbeSet was more accurate and directly averaged the F statistics. The kidney study divided genes into three overlapping groups: those showing mRNA abundances dependent on diet, gender, and strain. For all genes in those three groups that could be mapped to our dataset, we extracted their F-statistics and summarized them into boxplots (Figure 8A). Genes showing strain-dependent mRNA abundances in rat kidney showed a slight, but statistically-insignificant trend towards higher F-statistics in our liver dataset.

These data are limited by the use of different types and number of strains used in the two studies. Nevertheless, they do suggest that strain-dependent mRNA profiles will be tissue-specific. Further studies will be needed to address this question rigorously.

### Cross-Species Conservation of Inter-Strain Variability

The extent of inter-strain variability in hepatic mRNA levels is very large: hundreds of genes display inter-strain variability. This variability is non-random, as demonstrated by the clustering of biological replicates, the functional coherency of the set of perturbed genes, and the identification of specific TFBSs enriched in each strain-to-strain comparison. Further, analysis of the two lines descended from L-E×H/W crosses indicates that these differences are largely hereditary, with the vast majority following directional genetics.

Given this broad set of similarities, we hypothesized that similar sets of genes would display inter-strain variability in rat and its close rodent relative, mouse. To test this hypothesis we downloaded data from a previously published survey of steady-state hepatic mRNA levels in five strains of mouse [21]. To ensure optimal matching of homologs between these two species, we re-annotated each gene on the mouse array by mapping the provided GenBank accession IDs directly to a current UniGene build (Mm.168). We then employed the Homologene database to identify rat and mouse homologs. In total, 1332 matches between array elements were identified, representing 1018 unique pairs of genes.

We first compared the reported mouse Q-values and our own rat P-values (Figure 8B). Surprisingly, no association was observed (Spearman's rho = −0.04, p = 0.22). We repeated this analysis on the F-statistics and, again, did not see an association (data not shown). To ensure that the presence of multiple matches to each gene was not confounding our analysis we collapsed replicate ProbeSets in three different ways: by taking the mean, the minimum, and the maximum. The correlation analysis was repeated for each of these three datasets, and again no association was observed (data not shown). We conclude that genes that display inter-strain variability in the mouse are not more likely to display inter-strain variability in the rat, at least across the five mouse strains and five rat strains/lines considered in these two studies. Interestingly, the published mouse study found only 1.25% (66 of 5,285) of the transcripts on their array to be variable across strains at a 10% pFDR. By contrast, using an FDR threshold two orders of magnitude more stringent (0.1%), we identified three times more inter-strain variability (733/15,923 = 4.6%).

## Discussion

### Intra-Strain Variability

The variability in mRNA abundances amongst individuals has been the subject of several previous studies [8], [9], [21], [37] in other species, but previously only one limited study (with no public data) has addressed this question in the rat [38]. Inter-individual variability is a major confounding variable in clinical studies [39], [40], [41], thus the better we understand this phenomenon, the better we will be able to control for it. To address this issue, we introduced a mixed-modelling method, previously used successfully in oncogenomic studies, for comparing the variance within and amongst populations [24]. We show that a large number of genes display inter-individual variability and that the ratio of inter-individual to inter-strain variability is strongly related to gene function (Figure 2B). This finding corroborates that from an earlier study of Fischer F344 rats, where 8,833 genes were differentially expressed in at least one rat [38]. It is important to note that, by itself, this analysis says nothing about the magnitude of variance, only about how it is partitioned within individuals and strains. To address the limitations of the mixed-model analysis, we also verified these results using two independent methods: unsupervised clustering and linear-modelling.

Taken together, these data suggest that a large number of genes exhibit small-magnitude intra-strain variability. Our analysis could be strengthened by the use of technical replicates, to allow modelling of the variability associated with animal dissection, RNA extraction, and microarray hybridization. However all of these sources of variability would exaggerate intra-strain variability, making our conclusions a lower-bound on inter-strain heterogeneity. Further, it is important to note that all analyses presume that members of the populations are genetically identical. This is not strictly the case for the one out-bred (S-D) and the one closed-colony (H/W) strain used here. However, S-D and H/W rats are very widely used, and the lack of genetic identity would heighten, rather than reduce intra-strain variability, making our conclusions conservative. We also note that in the previous study of F344 rat, the authors suggest that less than 1% of genes show two-fold changes, corresponding to ∼88 genes – a similar number to that observed here [38].

### Inter-Strain Variability

Inter-strain variability affects, on average, 538 ProbeSets between each pair of strains/lines in this study. This is an underestimate of the overall inter-strain variability because the two lines used here are closely related to their parental strains.

With such large inter-strain variability, it is critical to understand if these genes are randomly distributed across the genome or if, instead, they are biased to specific locations and functions. We found that genes that exhibit inter-strain variability are localized into “islands” in the genome (Figure 4), which may reflect trends in allelic heterogeneity or copy-number variation [42]; large variations in copy-number exist between different inbred mouse strains [43]. Further, specific functional groups are enriched in each strain (Figure 5), increasing the probability that the observed expression differences will have large phenotypic effects. We considered one possible mechanism for these expression differences by performing a library-based search for transcription-factor binding-sites. Multiple transcription-factor motifs were found to be enriched in a combinatorial fashion across these strains.

Thus our results suggest that variability in hepatic mRNA abundances may cause some of the known phenotypic differences among strains. This variability may be caused in part by copy-number variation and in part by altered transcription-factor activities, although the relative contribution of these factors remains to be determined.

### AHR-Dependent Effects

It would be of great interest to know which single-nucleotide and copy-number polymorphisms are present in each of the animals of each strain/line used in this study so that we could comprehensively estimate their effect on mRNA abundances. Such a study is currently prohibitively expensive. Therefore, we focused on the one genomic locus whose status was known and variant amongst the five strains/lines: the aryl hydrocarbon receptor (*Ahr*) locus. Our analysis of mRNA changes associated with the Ahr locus provides an exemplar of how multi-strain, multi-replicate transcriptomic data can be used to assess the functional impact of specific polymorphisms. It would be of clear interest and utility to expand this analysis to groups of strains with varying genotypes of other critical pharmacological or toxicological genes, especially genes encoding nuclear receptors that regulate transcription such as CAR, GR, LXR and PXR.

The AHR is a major xenobiotic sensor and also plays important role in normal physiology [27], [28], [29], [44]. Previous work by our group showed that an aberrant form of the AHR leads to a profoundly reduced sensitivity to many dioxin-induced toxicities [22], [30], [45]. Two of the strain/lines used here (H/W and LnA) harbour the aberrant *Ahr*, while the remaining three do not. We used linear-modelling to identify *Ahr*-dependent changes in mRNA abundance and identified 105 ProbeSets showing significant associations with *Ahr* status at a 5% false-discovery rate. To determine if these changes were the effects of other *cis*-acting loci linked with the *Ahr* during recombination, we looked at their genomic distribution. None of the 105 ProbeSets were located in close proximity to the *Ahr* locus itself, and no “islands” of expression were identified that might indicate recombination-mediated effects. Accordingly, our results appear to reflect genuine AHR-mediated changes in abundance.

The gene whose transcript level was most greatly affected by AHR genotype was angiotensin II receptor type I (*Agtr1a*); levels were more than 50-fold higher in rats that have a deletion in the AHR transactivation domain than in rats with wildtype AHR (Table 1). The angiotensin II receptor plays a key role in regulation of blood pressure. Interestingly, if the AHR is knocked out in mice, plasma levels of angiotensin II, the main ligand for the angiotensin II receptor, rise 9-fold [46]. Further, angiotensin II is elevated in *Ahr*-null mice, and the AHR-associated gene *Bmal1* lies within a hypertension-susceptibility locus [47]. These observations, when considered in combination with the elevation of angiotensin II receptor in rats with an altered AHR transactivation domain, suggests that one physiologic function of the AHR is to suppress activity of the angiotensin II system and reduce the potential for hypertension and subsequent cardiac hypertrophy and fibrosis [46].

### Comparison of Rat and Mouse Inter-Strain Variability

Our experiments clearly show that inter-strain variability is a major phenomenon in the rat liver, involving hundreds of genes. Mechanistically it may be driven in part by copy-number variation or other regional genomic factors, and in part by differences in transcription-factor activities. Additional factors likely play a role, including epigenetic variations in methylation patterns or histone-modifications. We sought to determine if those genes displaying inter-strain variability in rat would be predisposed to it in another, closely related species, the mouse.

Upon mining a public dataset [21], we found no association between inter-strain variability in the two species. Formally, this may be a result of technical artifacts such as circadian effects [48], the small number of strains/lines used in each study (five), or the use of somewhat different statistical models. A more thorough and systematic cross-species study, incorporating technical replication, will be required to fully address these concerns. Nevertheless, our results provide an upper-bound on this variability and we believe that these confounding factors are minor. First, our study identified hundreds of genes, yielding a large number of data-points to be compared between the two species. Even subtle effects can be inferred from such large datasets. Second, while the statistical models are different, they are highly related, and in each study the specific genes in question were validated in multiple ways.

If strain-variability is not evolutionarily conserved, then this suggests that no single strain will be optimally representative of humans. Rather, to appropriately model human variability it may be most efficient to survey a panel of diverse strains. The techniques used here might be usefully employed to select this panel by identifying the most diverse representatives to be included.

## Methods

### Ethics

All animal study plans were approved by the Animal Experiment Committee of the University of Kuopio and the Provincial Government of Eastern Finland (Approval ID: ESLH-2008-07223/Ym-23).

### Animal Handling

Three rat strains/lines harbouring wildtype *Ahr* were selected: Sprague-Dawley (S-D), Long-Evans (*Turku/AB*) (L-E), and Line-C (LnC). Two rat strains/lines harbouring mutant AHR were selected: Han/Wistar (*Kuopio*) (H/W) and Line-A (LnA). Background information on the H/W and L-E strains can be found elsewhere [49]. The mutant AHR has been described elsewhere [30], as have the Line-A and Line-C strains [22]. Four animals of each strain were obtained from the breeding colonies of the National Public Health Institute, Division of Environmental Health, Kuopio, Finland; they were fed and housed under identical conditions in this facility. All animals were males 10–12 weeks old. Liver was harvested between 8:30 and 11:00 from rats treated by gavage with corn oil vehicle for 19 hours. The dose of corn oil was 4 mL/kg, which corresponds to approximately 15% of the daily calorie intake of the rats. The oral gavage procedure may have introduced some modest changes in mRNA expression [50]. Total RNA was extracted from both rat and mouse livers using Qiagen RNeasy kits according to the manufacturer's instructions (Qiagen, Mississauga, Canada). Total RNA yield was quantified by UV spectrophotometry and RNA integrity was verified using an Agilent 2100 Bioanalyzer (Agilent Technologies, Santa Clara, CA).

### mRNA Quantitation by Real-Time PCR

Total RNA (2 µg) was reverse-transcribed into cDNA using oligo-dT primer p(dT)15 (Roche Applied Science, Laval, QC, Canada) and Superscript II RNA polymerase according to the manufacturer's instructions (Invitrogen, Carlsbad, CA). Real-time PCR was performed on an MX4000 system (Stratagene, La Jolla, CA) using in-house designed primers and 5′fluorogenic probes to amplify from 250 ng of cDNA, as described elsewhere [51] and using Applied Biosystems gene expression assays to amplify from 100 ng of cDNA as described by the manufacturer (Applied Biosystems, Forest City, CA). Table S7 provides sequences for all primers/probe sets used.

Normalized expression (NE) was calculated using NE = 2^−ΔΔCt^, where C_t_ is the threshold cycle to detect fluorescence. PCR amplification efficiency was determined from a 10-fold serial dilution of a pool of cDNA; efficiency ranged from 90–110% for all genes examined. The data were normalized to either Actb or Gapdh.

For each gene, the PCR measurements were compared between all pairs of strains using a two-tailed t-test with Welch's adjustment for heteroscedasticity. Fold-changes were calculated, then log_2_-transformed for plotting.

### Pre-Processing and Statistical Analysis of Microarray Data

Affymetrix RAE230A arrays were run according to manufacturer's protocols at The Centre for Applied Genomics at The Hospital for Sick Children (Toronto, Canada). Four independent biological replicates (separate animals) were run for five strains of rat – Long-Evans, Han/Wistar, Sprague-Dawley, Line-A, and Line-C – for a total of 20 arrays. The raw array data have been deposited into the GEO repository with accession: GSE10448.

Microarray data were loaded into the R statistical environment (v2.6.1) using the affy package (v2.12.0) [52]. These data were pre-processed using the GC-RMA version of the RMA pre-processing algorithm [53], as implemented in the gcrma package (v2.10.0). Data were investigated for spatial and distributional homogeneity.

All clustering analyses employed divisive hierarchical clustering using the DIANA algorithm as implemented in the cluster package (v1.19.9) and with Pearson's correlation as a similarity metric. Heatmaps were visualized using the lattice (v0.17-4) and latticeExtra (v0.3-1) packages. Clustering was either based on global variance thresholds (per ProbeSet, across all 20 animals) or on global F-statistic thresholds (see below).

Intra-strain variability was assessed using the ratio of the within-strain variance to the total variance, as described previously [24]. Binning of samples by signal intensity was done separately for each strain.

Model-based t-tests were fit using the limma software package (v2.12.0) and subjected to an empirical Bayes moderation of the standard error [25]. P-values from this analysis were corrected for multiple testing with false-discovery rate adjustment [54]. Two separate linear models were fit:
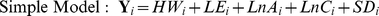


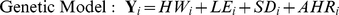



In the simple model, each strain/line is represented as a separate term in the linear model fit. A contrast matrix corresponding to all pair-wise comparisons was extracted, and the global F-statistic was used as a proxy for inter-strain variability in mRNA levels.

In the complex model, the proportion of the three parental strains is used to set the coefficients in the linear model fit. Thus the Han/Wistar arrays have a value of 1.0 for the H/W coefficient, and 0.0 for the L-E and S-D coefficients. The LnA and LnC animals receive values of 0.5 for the H/W and L-E coefficients, and 0.0 for the S-D coefficient. The value of the AHR coefficient is set as 1.0 if the animal harbours a variant AHR and 0.0 if the animal harbours a wild-type AHR.

For both models, we set our significance threshold at p_adjusted_<0.05. For the simple model we assessed threshold sensitivity by varying the p-value threshold in log steps from 10^−1^ to 10^−7^ and calculated the number of differentially-expressed ProbeSets at each value.

To identify genes with evidence for interacting loci [15] we focused on those ProbeSets with statistically significant (p_adjusted_<0.05) differential expression between Long-Evans and Han/Wistar rats. For each such ProbeSet we calculated the distance between Line-A and Line-C rats and the two parental strains using:
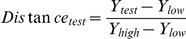
Where “test” is either Line-A or Line-C, Y indicates the normalized signal intensity, and “high” refers to either Long-Evans or Han/Wistar rats depending on which has *higher* signal intensity, and “low” refers to either Long-Evans or Han/Wistar rats depending on which has *lower* signal intensity. Gaussian kernel densities were fit to the distance values in the R statistical environment (v2.6.1). To identify specific ProbeSets displaying strong evidence of interacting loci we used the simple model described above. We selected those ProbeSets where the normalized signal intensity of Line-A or Line-C rats was significantly different (p_adjusted_<0.05) from both the parental strains with identical coefficient signs. That is, cases where Line-A or Line-C expression lay outside both of the parental strains.

Genes were annotated with version na24 of the Affymetrix NetAffx annotation (http://www.affymetrix.com). Genomic localization of AHR-responsive genes was performed using the geneplotter (v1.16.0), annotate (v1.16.1), and rae230a (v2.0.1) packages, all in version 2.6.1 of the R statistical environment.

### Transcription-Factor Binding-Site Analysis

We used a library-based method to search for transcription-factor binding-sites enriched or depleted in specific gene sets [55]. Using the CLOVER software package [56] we queried a 2005 version of the JASPAR database containing 123 position-weight matrices [57]. To ensure that our results were robust, five separate permutation tests were used. We employed simple mononucleotide and dinucleotide randomization as well as randomization of the position-weight matrices themselves. Additionally, two background datasets were used. The first contained the promoters of all genes present on the RAE230A array and the second contained the promoters of all putatively expressed genes. Expressed genes were identified as those with an average normalized signal intensity of 4 or greater. This threshold was derived by analyzing the expression of y-chromosome genes in females [24]. In total 5042 of the 9735 unique Entrez Gene IDs on the RAE230A array were “expressed” at this threshold. For each permutation test 1,000 randomizations were performed with a p-value threshold of 0.05 and a scores threshold of 5. Only motifs significantly enriched or depleted in at least four tests are reported. We note that for highly repetitive matrices, randomization of the matrix columns will be biased against true signals. Genomic sequences from −1,000 to +1,000 relative to the transcriptional-start site were used, for sequences of 2,001 bp. These sequences were extracted from build rn4 of the rat genome using annotation from the UCSC genome browser database downloaded on 2007-04-07 [58].

### Gene Ontology Enrichment analysis

Functional enrichment analysis was performed using the GOMiner tool [36]. All rat databases, look-up options, ontologies, and evidence levels were included. False-discovery rates were estimated with 1,000 randomizations and a 10% FDR threshold was set. We formed the matrix of GO-terms by contrasts using all pair-wise contrasts from the simple model and the AHR term from the complex model. The log_10_(P) values for each GO-term were summed. Subsets of the matrix containing those GO terms with sums of at least 5, 7.5, 10, 20, and 30 were generated. These subsets were clustered using Pearson's correlation as the distance metric and the DIANA divisive hierarchical clustering algorithm as implemented in the cluster package (v1.11.9) for the R statistical environment (v2.6.1). The clustering patterns were visualized using heatmaps as generated by the lattice (v0.17-4) and latticeExtra (v0.3-1) packages.

### Comparison of AHR Genes With Public Data

Genes showing basal differences in mRNA abundance between strains bearing different *AHR* alleles were further compared to public datasets. First, we analyzed microarray data on the effects of ligand stimulation in rat liver. A dataset studying the response to TCDD of four rat strains in our study (H/W, L-E, Line-A, and Line –C) was analyzed [31]. ProbeSets were mapped between the two studies based on Entrez Gene IDs, and only those statistically significant in either dataset (p_adjusted_<0.05) were retained. The magnitude of the response to TCDD was then plotted against the magnitude of the response to variant AHR alleles. The Pearson correlation between these two variables was calculated. Second, we analyzed microarray data on the effects of both *AHR* genotype and ligand stimulation in mouse liver [32]. ProbeSets were mapped separately to Entrez Gene IDs for the rat and mouse datasets, and then merged using the Homologene database (build 64). For ProbeSets that were statistically significant in either dataset (p_adjusted_<0.05) the magnitude of the response to variant rat alleles in the current study was plotted against the magnitude of the mouse response to TCDD and against the magnitude of the effect of ablation of the *AHR* locus in mice. Again, Pearson's correlation was calculated between each pair of variables. These analyses were performed in the R statistical environment (v2.9.2) and used the lattice package (v0.17-26) for plotting.

### Kidney-Liver Comparative Analysis

To contrast inter-strain variability in two rat tissues, we downloaded the supplementary data from Seidel et al. [18]. We updated the annotation of their microarray by directly matching each GenBank accession ID to a UniGene cluster and Entrez Gene ID using UniGene build Rn.171. We then matched genes between our simple pair-wise comparison analysis and their dataset using Entrez Gene IDs. We extracted the F-statistic for each matching gene. In cases where multiple ProbeSets existed for a single gene, we averaged the F statistics for all ProbeSets to avoid biasing our analysis. Boxplots of the F-statistics in log_2_-space were created for each subset predefined by Seidel and co-workers [18], as well as for all genes matching between the two arrays. Two-tailed unpaired t-tests were used for statistical analysis, with Welch's adjustment for heteroscedasticity. All analyses were performed in the R statistical environment (v2.6.2).

### Rat-Mouse Comparative Analysis

To compare inter-strain variability between rat and mouse we downloaded the supplementary data from Pritchard et al. [21]. We updated the annotation of their microarray by directly matching each GenBank accession ID to a UniGene cluster and Entrez Gene ID using UniGene build Mm.168. We then matched the mouse genes to their rat homologs using build 58 of the Homologene database. Correlation analyses used Spearman's rho. To account for cases where multiple rat ProbeSets existed for a single murine gene, we repeated our analysis with unaggregated data, data aggregated by taking the mean F-statistic across ProbeSets, data aggregated by selecting the minimum F-statistic across ProbeSets, and data aggregated by selected the maximum F-statistic across ProbeSets.

## Supporting Information

Figure S1 Agglomerative hierarchical clustering of un-pre-processed data.
(PPT)

Figure S2 RNA degradation plots for all arrays in experiment.
(PPT)

Figure S3 Density plot of all Probes prior to normalization.
(PPT)

Figure S4 Divisive hierarchical clustering of expression data using an F-statistic filter.
(PPT)

Figure S5 Clustering of Gene Ontology data at P_cumulative_<10^−5^.
(PPT)

Figure S6 Clustering of Gene Ontology data at P_cumulative_<10^−7.5^.
(PPT)

Figure S7 Clustering of Gene Ontology data at P_cumulative_<10^−20^.
(PPT)

Figure S8 Clustering of Gene Ontology data at P_cumulative_<10^−30^.
(PPT)

Table S1 Complete W/T analysis for all ProbeSets. The within-strain (W), between-strain (B), and total (T) variances are given for every ProbeSet, along with the mean signal intensity, the expression quartile (high, medium, low, unexpressed) and the ratio W/T.
(XLS)

Table S2 Complete table of log_10_ p-values for enrichment of all GO terms for all W/T groups. Each decile of W/T (i.e. 0.0 to 0.1) is listed as a column, and the rows correspond to all GO terms represented by genes on the microarray. Each cell gives a log_10_|P| value for the enrichment (Fisher's Exact test) of that GO term in genes in that decile of W/T.
(XLS)

Table S3 Complete linear model fit for all coefficients using simple pair-wise model. For all ProbeSets on the microarray (rows) a number of database annotations are given (UniGene Cluster, Gene Name and Symbol, Entrez Gene ID, SwissProt Accession). A series of ten columns gives the multiple-testing adjusted p-value for differential expression of each ProbeSet in each pair of rat strains/lines.
(XLS)

Table S4 Complete linear model fit for all coefficients using AHR-dependent model. For all ProbeSets on the microarray (rows) a number of database annotations are given (UniGene Cluster, Gene Name and Symbol, Entrez Gene ID, SwissProt Accession). The average signal intensity (A) is also given, followed by the coefficients of each strain and of the AHR effect. The t-statistics and naïve and multiple-testing adjusted p-values are also given for each term in the model. Finally, the F statistic and p-value for the entire model are shown for each ProbeSet.
(XLS)

Table S5 Complete table of log_10_ p-values for enrichment of all GO terms for all contrasts. For each contrast from the strain-wise linear-model fit (columns) the p-values for enrichment of each GO term (rows) are shown in log_10_-space.
(XLS)

Table S6 Table of all interacting loci identified. A list of the 48 ProbeSets/lines that show evidence of interaction, with the line exhibiting signal intensities outside either of its parental strains. The Gene Symbol, Name, Entrez Gene ID, and order of strains is given for each.
(XLS)

Table S7 List of all primer and probe sequences used in real-time PCR experiments.
(XLS)

## References

[1] Amin RP, Hamadeh HK, Bushel PR, Bennett L, Afshari CA (2002). Genomic interrogation of mechanism(s) underlying cellular responses to toxicants.. Toxicology.

[2] Burgoon LD, Boutros PC, Dere E, Zacharewski TR (2006). dbZach: A MIAME-compliant toxicogenomic supportive relational database.. Toxicol Sci.

[3] Guo L, Lobenhofer EK, Wang C, Shippy R, Harris SC (2006). Rat toxicogenomic study reveals analytical consistency across microarray platforms.. Nat Biotechnol.

[4] Jacob HJ, Kwitek AE (2002). Rat genetics: attaching physiology and pharmacology to the genome.. Nat Rev Genet.

[5] Lazar J, Moreno C, Jacob HJ, Kwitek AE (2005). Impact of genomics on research in the rat.. Genome Res.

[6] Martin R, Rose D, Yu K, Barros S (2006). Toxicogenomics strategies for predicting drug toxicity.. Pharmacogenomics.

[7] Frijters R, Verhoeven S, Alkema W, van Schaik R, Polman J (2007). Literature-based compound profiling: application to toxicogenomics.. Pharmacogenomics.

[8] Jin W, Riley RM, Wolfinger RD, White KP, Passador-Gurgel G (2001). The contributions of sex, genotype and age to transcriptional variance in Drosophila melanogaster.. Nat Genet.

[9] Ranz JM, Castillo-Davis CI, Meiklejohn CD, Hartl DL (2003). Sex-dependent gene expression and evolution of the Drosophila transcriptome.. Science.

[10] Fernandes C, Paya-Cano JL, Sluyter F, D'Souza U, Plomin R (2004). Hippocampal gene expression profiling across eight mouse inbred strains: towards understanding the molecular basis for behaviour.. Eur J Neurosci.

[11] Daniels GM, Buck KJ (2002). Expression profiling identifies strain-specific changes associated with ethanol withdrawal in mice.. Genes Brain Behav.

[12] Cheung VG, Conlin LK, Weber TM, Arcaro M, Jen KY (2003). Natural variation in human gene expression assessed in lymphoblastoid cells.. Nat Genet.

[13] Morley M, Molony CM, Weber TM, Devlin JL, Ewens KG (2004). Genetic analysis of genome-wide variation in human gene expression.. Nature.

[14] Cheung VG, Spielman RS, Ewens KG, Weber TM, Morley M (2005). Mapping determinants of human gene expression by regional and genome-wide association.. Nature.

[15] Rockman MV, Kruglyak L (2006). Genetics of global gene expression.. Nat Rev Genet.

[16] Monks SA, Leonardson A, Zhu H, Cundiff P, Pietrusiak P (2004). Genetic inheritance of gene expression in human cell lines.. Am J Hum Genet.

[17] Schadt EE, Monks SA, Drake TA, Lusis AJ, Che N (2003). Genetics of gene expression surveyed in maize, mouse and man.. Nature.

[18] Seidel SD, Hung SC, Lynn Kan H, Bhaskar Gollapudi B (2006). Background gene expression in rat kidney: influence of strain, gender, and diet.. Toxicol Sci.

[19] Cerutti C, Kurdi M, Bricca G, Hodroj W, Paultre C (2006). Transcriptional alterations in the left ventricle of three hypertensive rat models.. Physiol Genomics.

[20] Boedigheimer MJ, Wolfinger RD, Bass MB, Bushel PR, Chou JW (2008). Sources of variation in baseline gene expression levels from toxicogenomics study control animals across multiple laboratories.. BMC Genomics.

[21] Pritchard C, Coil D, Hawley S, Hsu L, Nelson PS (2006). The contributions of normal variation and genetic background to mammalian gene expression.. Genome Biol.

[22] Tuomisto JT, Viluksela M, Pohjanvirta R, Tuomisto J (1999). The AH receptor and a novel gene determine acute toxic responses to TCDD: segregation of the resistant alleles to different rat lines.. Toxicol Appl Pharmacol.

[23] Boutros PC, Okey AB (2005). Unsupervised pattern recognition: an introduction to the whys and wherefores of clustering microarray data.. Brief Bioinform.

[24] Bachtiary B, Boutros PC, Pintilie M, Shi W, Bastianutto C (2006). Gene expression profiling in cervical cancer: an exploration of intratumor heterogeneity.. Clin Cancer Res.

[25] Smyth GK (2003). Linear Models and Empirical Bayes Methods for Assessing Differential Expression in Microarray Experiments.. Stat App Genet Mol Biol.

[26] Gibbs RA, Weinstock GM, Metzker ML, Muzny DM, Sodergren EJ (2004). Genome sequence of the Brown Norway rat yields insights into mammalian evolution.. Nature.

[27] Okey AB, Riddick DS, Harper PA (1994). Molecular biology of the aromatic hydrocarbon (dioxin) receptor.. Trends Pharmacol Sci.

[28] McMillan BJ, Bradfield CA (2007). The aryl hydrocarbon receptor sans xenobiotics: endogenous function in genetic model systems.. Mol Pharmacol.

[29] Okey AB (2007). An aryl hydrocarbon receptor odyssey to the shores of toxicology: the Deichmann Lecture, International Congress of Toxicology-XI.. Toxicol Sci.

[30] Pohjanvirta R, Wong JM, Li W, Harper PA, Tuomisto J (1998). Point mutation in intron sequence causes altered carboxyl-terminal structure in the aryl hydrocarbon receptor of the most 2,3,7,8-tetrachlorodibenzo-p-dioxin-resistant rat strain.. Mol Pharmacol.

[31] Moffat ID, Boutros PC, Chen H, Okey AB, Pohjanvirta R (2010). Aryl hydrocarbon receptor (AHR)-regulated transcriptomic changes in rats sensitive or resistant to major dioxin toxicities.. BMC Genomics.

[32] Boutros PC, Bielefeld KA, Pohjanvirta R, Harper PA (2009). Dioxin-dependent and dioxin-independent gene batteries: comparison of liver and kidney in AHR-null mice.. Toxicol Sci.

[33] Tijet N, Boutros PC, Moffat ID, Okey AB, Tuomisto J (2006). Aryl hydrocarbon receptor regulates distinct dioxin-dependent and dioxin-independent gene batteries.. Mol Pharmacol.

[34] Boutros PC, Yan R, Moffat ID, Pohjanvirta R, Okey AB (2008). Transcriptomic responses to 2,3,7,8-tetrachlorodibenzo-p-dioxin (TCDD) in liver: comparison of rat and mouse.. BMC Genomics.

[35] Boverhof DR, Burgoon LD, Tashiro C, Sharratt B, Chittim B (2006). Comparative toxicogenomic analysis of the hepatotoxic effects of TCDD in Sprague Dawley rats and C57BL/6 mice.. Toxicol Sci.

[36] Zeeberg BR, Feng W, Wang G, Wang MD, Fojo AT (2003). GoMiner: a resource for biological interpretation of genomic and proteomic data.. Genome Biol.

[37] Pritchard CC, Hsu L, Delrow J, Nelson PS (2001). Project normal: defining normal variance in mouse gene expression.. Proc Natl Acad Sci U S A.

[38] Boorman GA, Irwin RD, Vallant MK, Gerken DK, Lobenhofer EK (2005). Variation in the hepatic gene expression in individual male Fischer rats.. Toxicol Pathol.

[39] Boutros PC, Lau SK, Pintilie M, Liu N, Shepherd FA (2009). Prognostic gene signatures for non-small-cell lung cancer.. Proc Natl Acad Sci U S A.

[40] van de Vijver MJ, He YD, van't Veer LJ, Dai H, Hart AA (2002). A gene-expression signature as a predictor of survival in breast cancer.. N Engl J Med.

[41] Chen HY, Yu SL, Chen CH, Chang GC, Chen CY (2007). A five-gene signature and clinical outcome in non-small-cell lung cancer.. N Engl J Med.

[42] Redon R, Ishikawa S, Fitch KR, Feuk L, Perry GH (2006). Global variation in copy number in the human genome.. Nature.

[43] Cutler G, Marshall LA, Chin N, Baribault H, Kassner PD (2007). Significant gene content variation characterizes the genomes of inbred mouse strains.. Genome Res.

[44] Gu YZ, Hogenesch JB, Bradfield CA (2000). The PAS superfamily: sensors of environmental and developmental signals.. Annu Rev Pharmacol Toxicol.

[45] Pohjanvirta R, Viluksela M, Tuomisto JT, Unkila M, Karasinska J (1999). Physicochemical differences in the AH receptors of the most TCDD-susceptible and the most TCDD-resistant rat strains.. Toxicol Appl Pharmacol.

[46] Lund AK, Goens MB, Kanagy NL, Walker MK (2003). Cardiac hypertrophy in aryl hydrocarbon receptor null mice is correlated with elevated angiotensin II, endothelin-1, and mean arterial blood pressure.. Toxicol Appl Pharmacol.

[47] Woon PY, Kaisaki PJ, Braganca J, Bihoreau MT, Levy JC (2007). Aryl hydrocarbon receptor nuclear translocator-like (BMAL1) is associated with susceptibility to hypertension and type 2 diabetes.. Proc Natl Acad Sci U S A.

[48] Boorman GA, Blackshear PE, Parker JS, Lobenhofer EK, Malarkey DE (2005). Hepatic gene expression changes throughout the day in the Fischer rat: implications for toxicogenomic experiments.. Toxicol Sci.

[49] Pohjanvirta R, Tuomisto J (1990). Mechanism of action of 2,3,7,8-tetrachlorodibenzo-p-dioxin (TCDD).. Toxicol Appl Pharmacol.

[50] Pohjanvirta R, Boutros PC, Moffat ID, Linden J, Wendelin D (2008). Genome-wide effects of acute progressive feed restriction in liver and white adipose tissue.. Toxicol Appl Pharmacol.

[51] Franc MA, Moffat ID, Boutros PC, Tuomisto JT, Tuomisto J (2008). Patterns of dioxin-altered mRNA expression in livers of dioxin-sensitive versus dioxin-resistant rats.. Arch Toxicol.

[52] Gautier L, Cope L, Bolstad BM, Irizarry RA (2004). affy–analysis of Affymetrix GeneChip data at the probe level.. Bioinformatics.

[53] Irizarry RA, Bolstad BM, Collin F, Cope LM, Hobbs B (2003). Summaries of Affymetrix GeneChip probe level data.. Nucleic Acids Res.

[54] Efron B, Tibshirani R (2002). Empirical bayes methods and false discovery rates for microarrays.. Genet Epidemiol.

[55] Wasserman WW, Sandelin A (2004). Applied bioinformatics for the identification of regulatory elements.. Nat Rev Genet.

[56] Frith MC, Fu Y, Yu L, Chen JF, Hansen U (2004). Detection of functional DNA motifs via statistical over-representation.. Nucleic Acids Res.

[57] Sandelin A, Alkema W, Engstrom P, Wasserman WW, Lenhard B (2004). JASPAR: an open-access database for eukaryotic transcription factor binding profiles.. Nucleic Acids Res.

[58] Karolchik D, Baertsch R, Diekhans M, Furey TS, Hinrichs A (2003). The UCSC Genome Browser Database.. Nucleic Acids Res.

