# Supplementary figures and images for: mRNA Levels in Control Rat Liver Display Strain-Specific, Hereditary, and AHR-Dependent Components
Source: PLoS One. 2011 Jul 8;6(7):e18337. doi: 10.1371/journal.pone.0018337 (PMC3132743; doi:10.1371/journal.pone.0018337)

## Slide 1
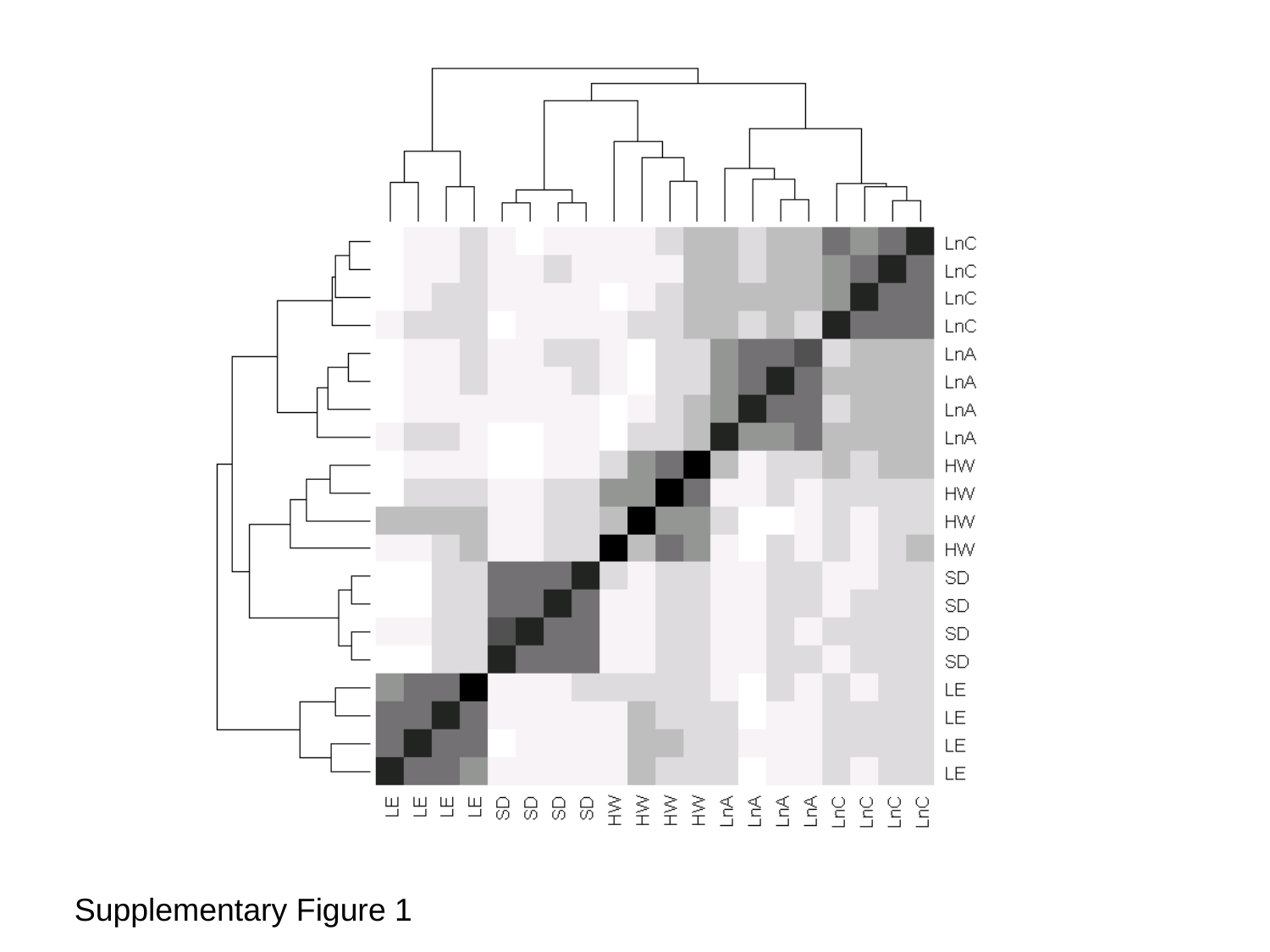

Supplementary Figure 1

Supplement: Figure S1 — Agglomerative hierarchical clustering of un-pre-processed data. (PPT) [file pone.0018337.s001.ppt]

## Slide 1
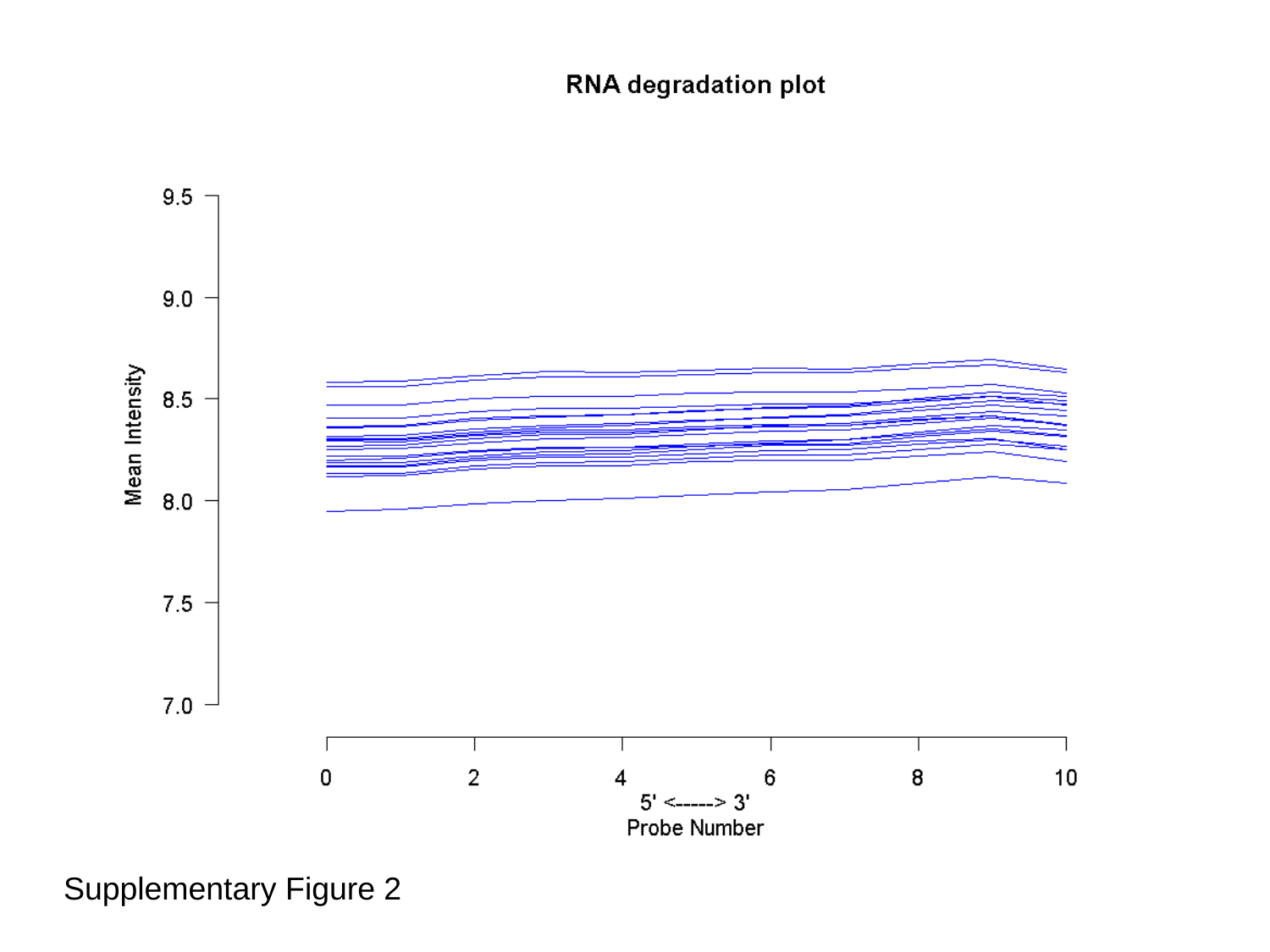

Supplementary Figure 2

Supplement: Figure S2 — RNA degradation plots for all arrays in experiment. (PPT) [file pone.0018337.s002.ppt]

## Slide 1
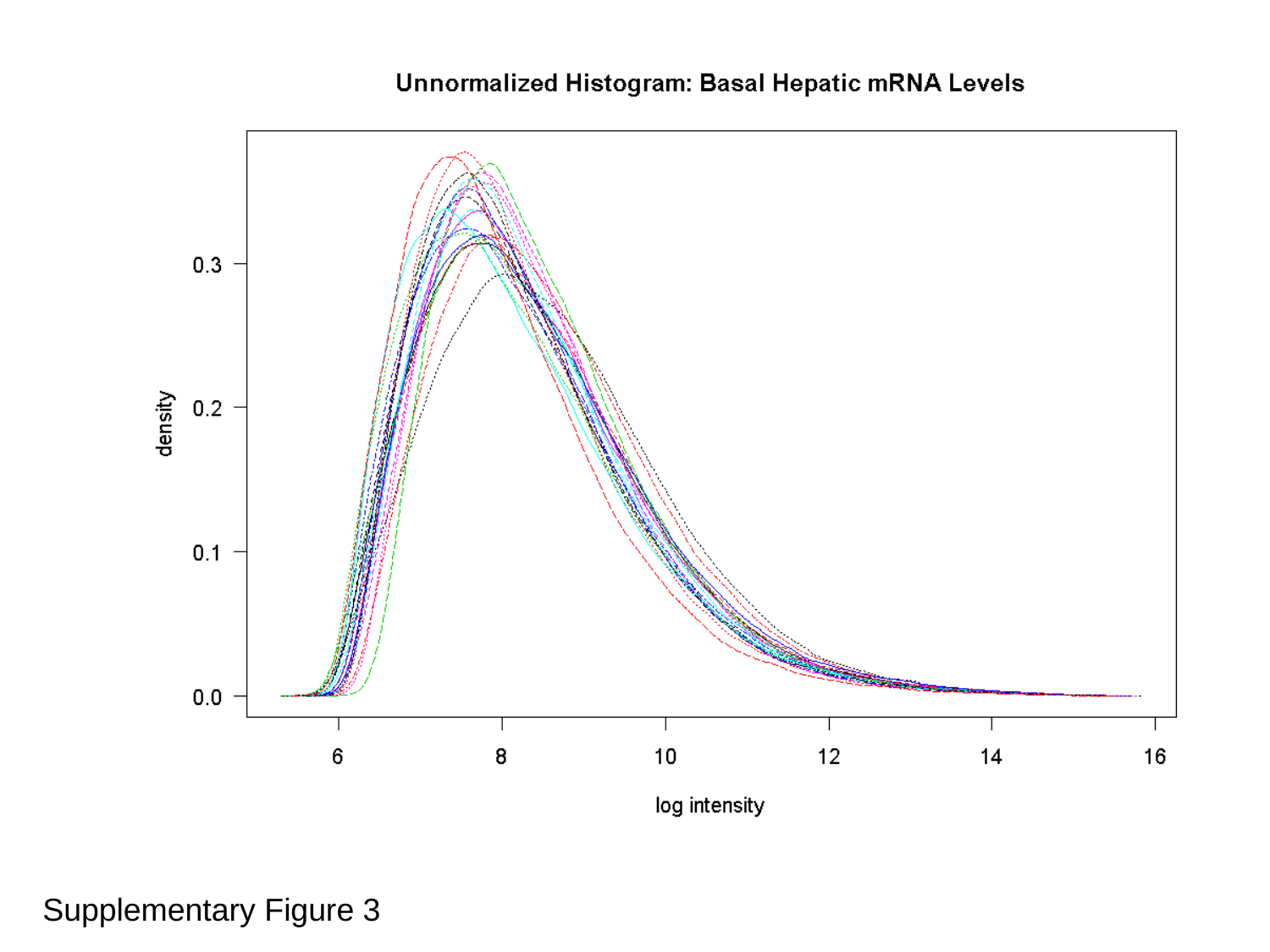

Supplementary Figure 3

Supplement: Figure S3 — Density plot of all Probes prior to normalization. (PPT) [file pone.0018337.s003.ppt]

## Slide 1
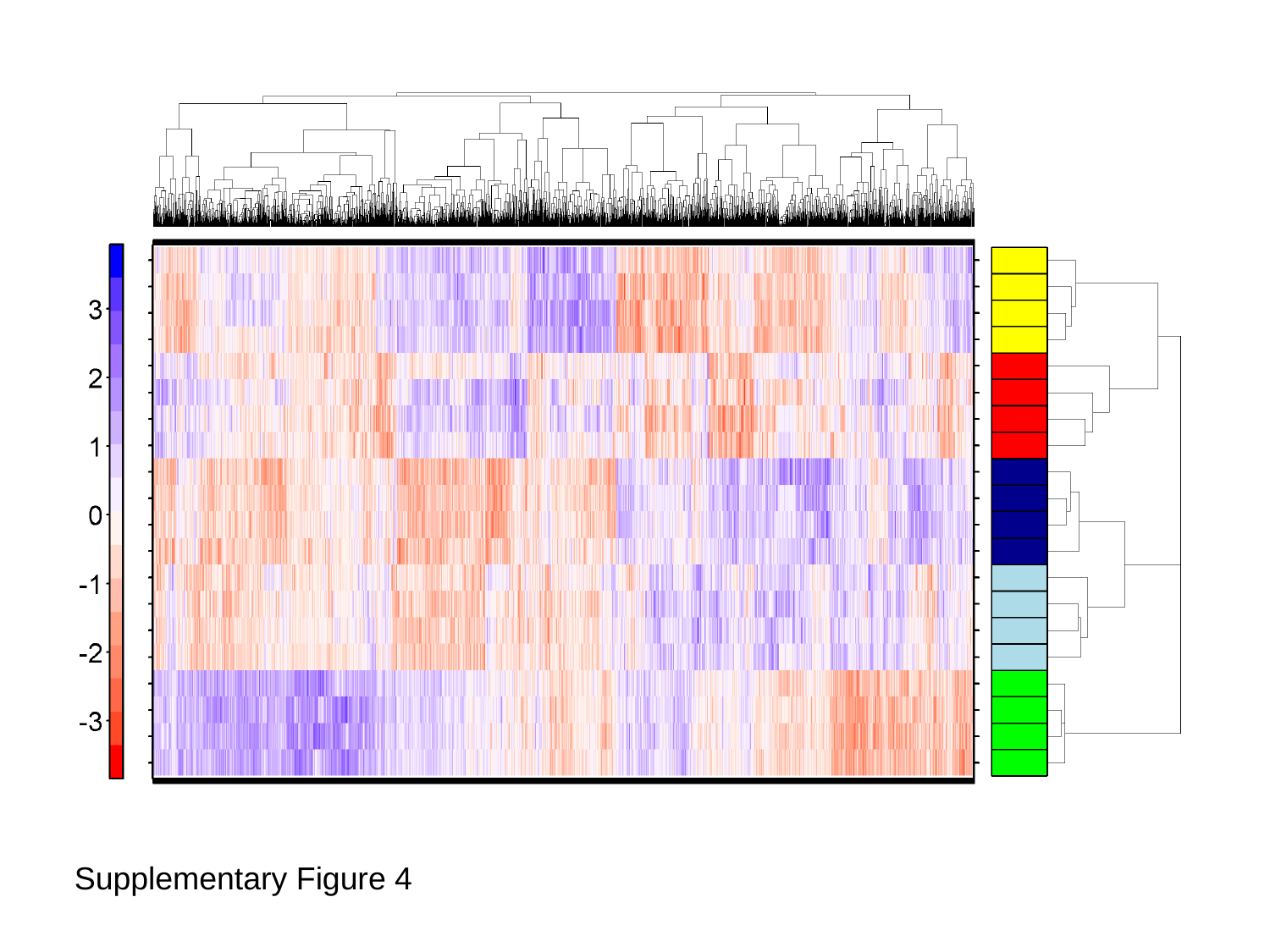

Supplementary Figure 4

Supplement: Figure S4 — Divisive hierarchical clustering of expression data using an F-statistic filter. (PPT) [file pone.0018337.s004.ppt]

## Slide 1
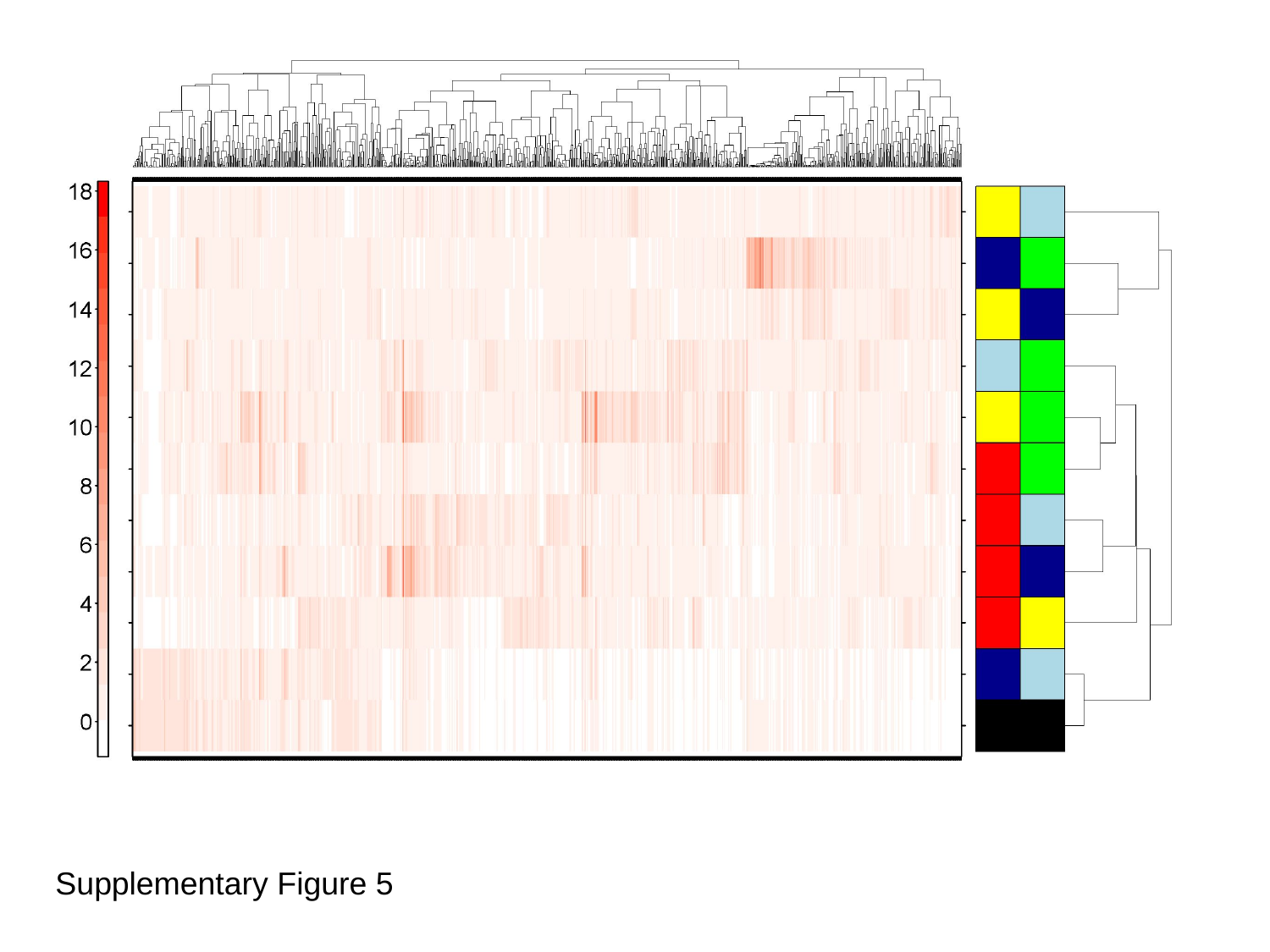

Supplementary Figure 5

Supplement: Figure S5 — Clustering of Gene Ontology data at Pcumulative<10−5. (PPT) [file pone.0018337.s005.ppt]

## Slide 1
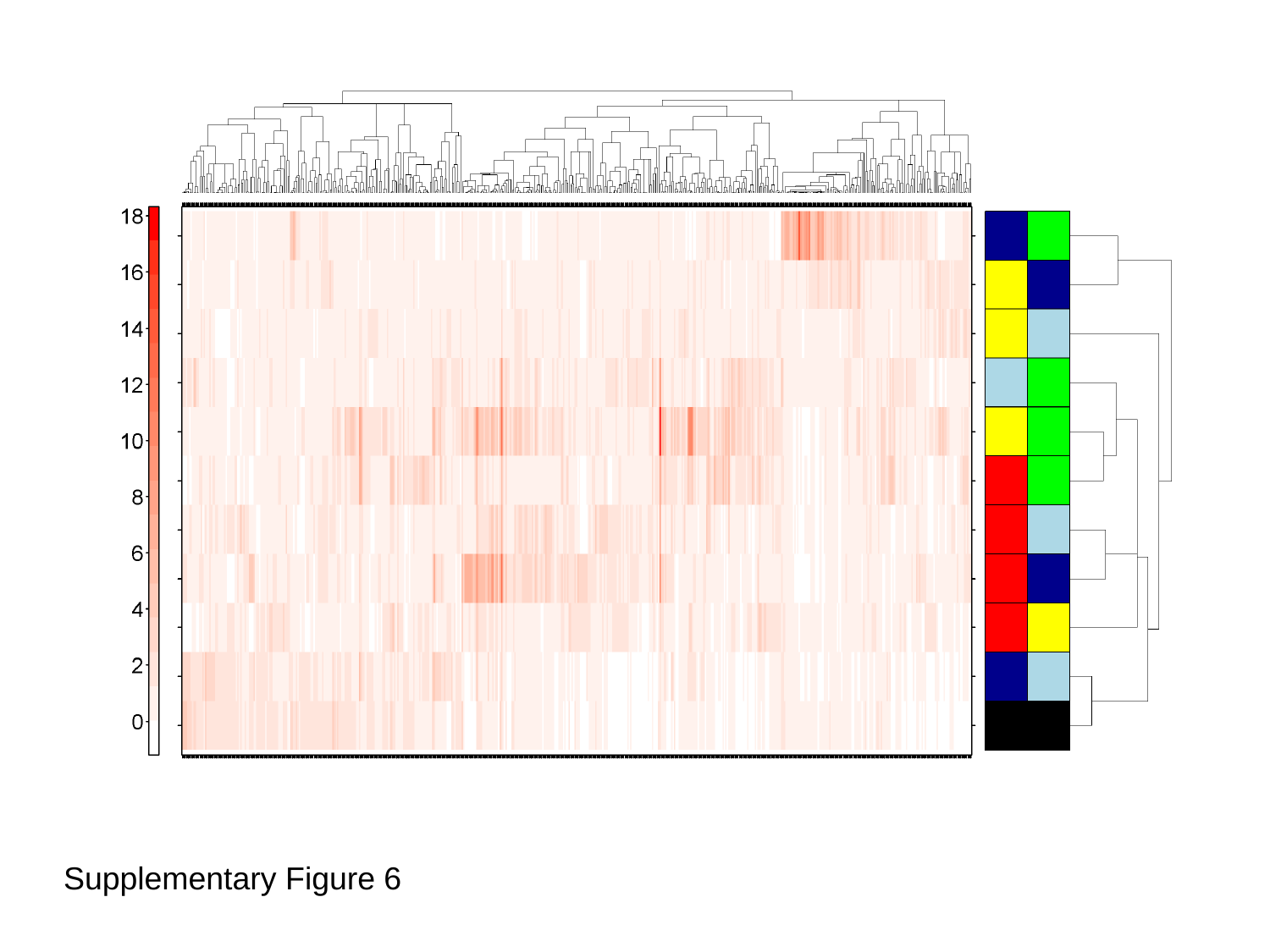

Supplementary Figure 6

Supplement: Figure S6 — Clustering of Gene Ontology data at Pcumulative<10−7.5. (PPT) [file pone.0018337.s006.ppt]

## Slide 1
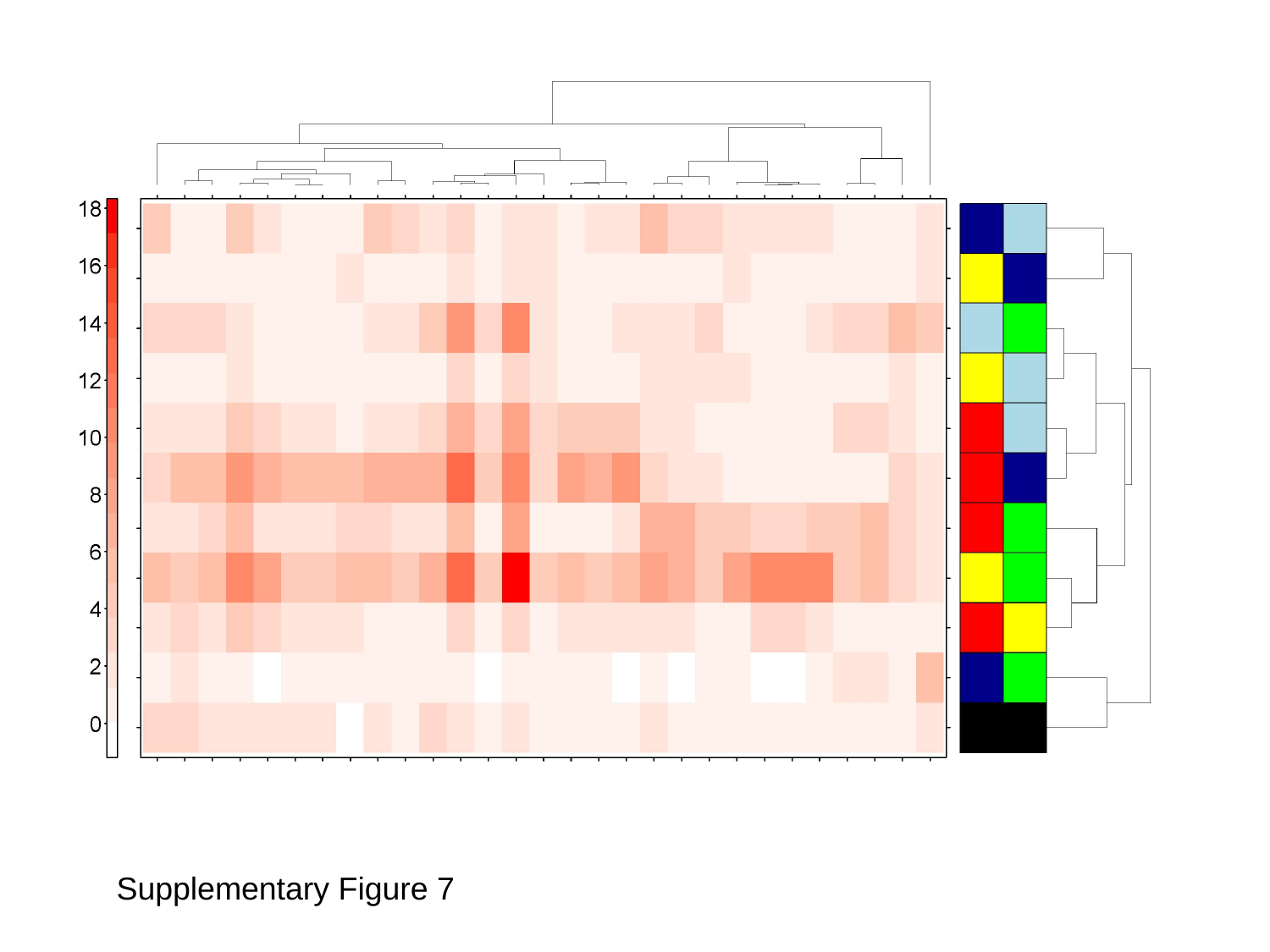

Supplementary Figure 7

Supplement: Figure S7 — Clustering of Gene Ontology data at Pcumulative<10−20. (PPT) [file pone.0018337.s007.ppt]

## Slide 1
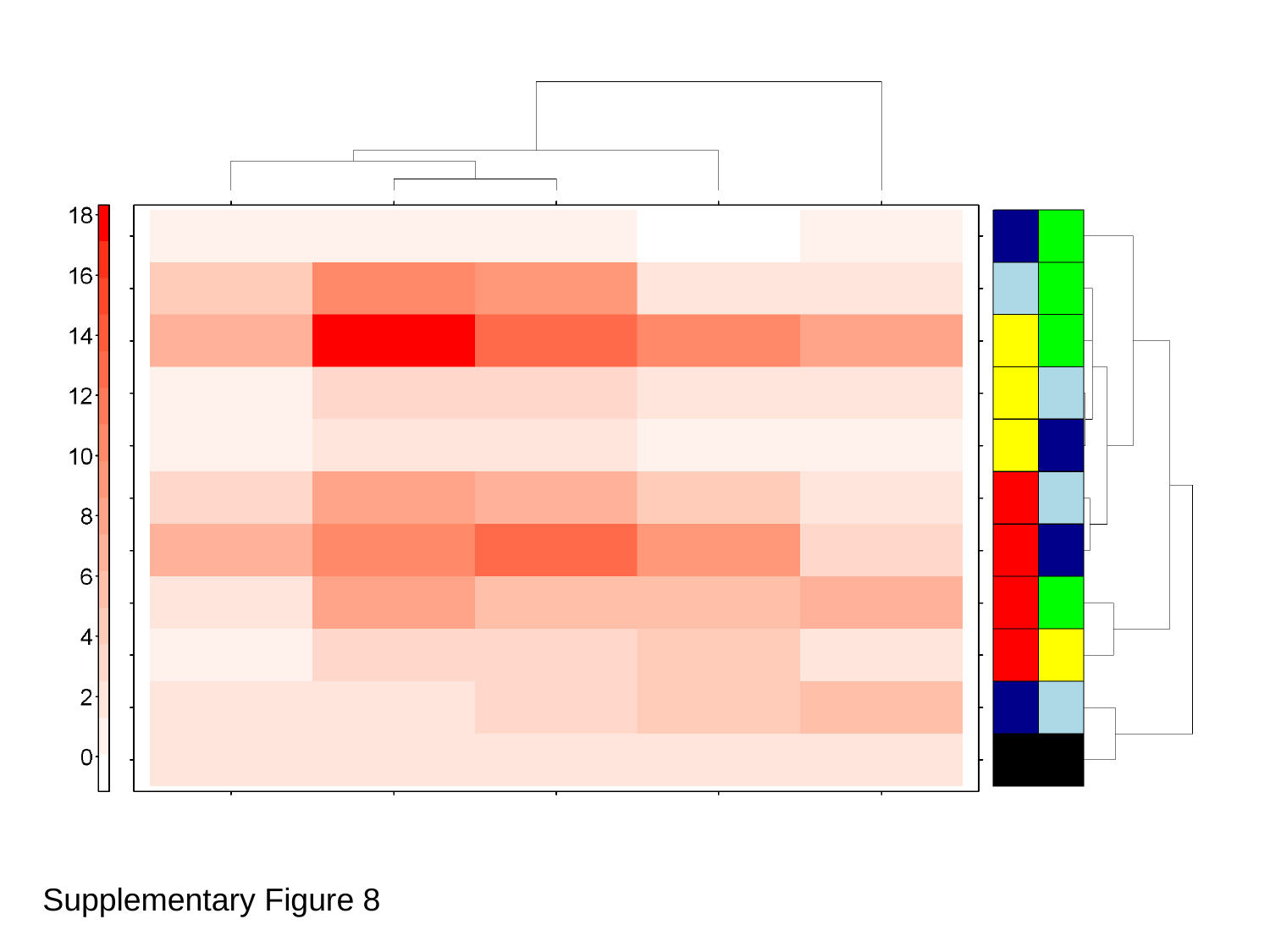

Supplementary Figure 8

Supplement: Figure S8 — Clustering of Gene Ontology data at Pcumulative<10−30. (PPT) [file pone.0018337.s008.ppt]
